# Welches Potential hat ChatGPT 3.5 für eine qualifizierte Patienteninformation? Versuch einer systematischen Analyse anhand einer Befragung zu komplementärmedizinischen Verfahren in der Rheumatologie

**DOI:** 10.1007/s00393-024-01535-6

**Published:** 2024-07-10

**Authors:** Gernot Keyßer, Alexander Pfeil, Monika Reuß-Borst, Inna Frohne, Olaf Schultz, Oliver Sander

**Affiliations:** 1 Klinik und Poliklinik für Innere Medizin II, Universitätsklinikum Halle, Ernst-Grube-Str. 40, 06120 Halle (Saale), Deutschland; 2 Klinik für Innere Medizin III, Universitätsklinikum Jena, Friedrich-Schiller-Universität Jena, Jena, Deutschland; 3 Facharztpraxis für Innere Medizin, Bad Bocklet, Deutschland; 4 Privatpraxis für Rheumatologie, Essen, Deutschland; 5 Abteilung Rheumatologie, ACURA Kliniken Baden-Baden, Baden-Baden, Deutschland; 6 Klinik für Rheumatologie, Universitätsklinikum Düsseldorf, Düsseldorf, Deutschland

**Keywords:** Künstliche Intelligenz, Entzündlich-rheumatische Erkrankungen, Ayurvedische Medizin, Homöopathie, Phytotherapie, Artificial intelligence, Inflammatory rheumatic diseases, Ayurvedic medicine, Homeopathy, Herbal medicine

## Abstract

**Einführung:**

Der Chatbot ChatGPT stellt einen Meilenstein in der Interaktion zwischen Menschen und großen, über das Internet zugänglichen Datenbanken dar. Er ermöglicht mit einer Kommunikation in Alltagssprache die Beantwortung komplexer Fragen und ist damit potenziell eine Informationsquelle für Betroffene rheumatischer Erkrankungen. Ziel der Untersuchung war es herauszufinden, ob ChatGPT (Version 3.5) in der Lage ist, qualifizierte Antworten zur Anwendbarkeit von Verfahren der Komplementär- und Alternativmedizin (CAM; Homöopathie, Ayurveda, Phytotherapie) bei rheumatoider Arthritis (RA), systemischem Lupus erythematodes (SLE) und Granulomatose mit Polyangiitis (GPA) zu liefern. Außerdem wurde untersucht, welchen Einfluss die Art der Fragestellung auf die erhaltenen Ergebnisse haben könnte.

**Methodik:**

Die Befragung erfolgte in 3 Abschnitten. In Abschnitt A wurde eine offene Frage zu Behandlungsmöglichkeiten bei einem der 3 Krankheitsbilder gestellt. In Abschnitt B wurde allgemein nach möglichen Anwendungen für CAM bei einer der 3 Erkrankungen gefragt. In Abschnitt C wurden Applikationsmöglichkeiten für die 3 genannten Verfahren für jede Diagnose erfragt. In den Abschnitten B und C wurden die Fragen jeweils in 2 Modifikationen gestellt. Die erste fragte danach, ob das Verfahren überhaupt anwendbar ist. Die zweite Frage erkundigte sich nach konkreten Anwendungen aus den genannten Verfahren. Die Validität der Ergebnisse wurde anhand des ChatGPT Reliability Scores, einer 7‑stufigen Likert-Skala, ausgewertet.

**Ergebnisse:**

Zu den offenen Fragen im Abschnitt A lieferte ChatGPT die validesten Ergebnisse. In B und C wurden zahlreiche CAM-Anwendungen vorgeschlagen, die nicht durch wissenschaftliche Evidenz gestützt sind. In diesen Abschnitten waren die Ergebnisse deutlich von der Art der Fragestellung abhängig. Suggerierte die Frage eine Anwendungsabsicht der CAM, entfielen häufig Hinweise auf die fehlende Evidenz, die Qualität der Antwort wurde in den meisten Fällen schlechter bewertet.

**Schlussfolgerung:**

Die Antworten von ChatGPT zur Anwendung von CAM bei definierten rheumatischen Erkrankungen lassen eine ausreichende wissenschaftliche Evidenz vermissen. Zudem beeinflusst die Art der Fragestellung die Qualität der Aussagen erheblich. Eine kritiklose Anwendung von ChatGPT als Instrument der Patientenschulung kann derzeit nicht empfohlen werden.

**Zusatzmaterial online:**

Die Online-Version dieses Beitrags (10.1007/s00393-024-01535-6) enthält das Protokoll der Chat-GPT-Antworten.

## Abstract (en)

**Introduction:**

The chatbot ChatGPT represents a milestone in the interaction between humans and large databases that are accessible via the internet. It facilitates the answering of complex questions by enabling a communication in everyday language. Therefore, it is a potential source of information for those who are affected by rheumatic diseases.

The aim of our investigation was to find out whether ChatGPT (version 3.5) is capable of giving qualified answers regarding the application of specific methods of complementary and alternative medicine (CAM) in three rheumatic diseases: rheumatoid arthritis (RA), systemic lupus erythematosus (SLE) and granulomatosis with polyangiitis (GPA). In addition, it was investigated how the answers of the chatbot were influenced by the wording of the question.

**Methods:**

The questioning of ChatGPT was performed in three parts. Part A consisted of an open question regarding the best way of treatment of the respective disease. In part B, the questions were directed towards possible indications for the application of CAM in general in one of the three disorders. In part C, the chatbot was asked for specific recommendations regarding one of three CAM methods: homeopathy, ayurvedic medicine and herbal medicine. Questions in parts B and C were expressed in two modifications: firstly, it was asked whether the specific CAM was applicable at all in certain rheumatic diseases. The second question asked which procedure of the respective CAM method worked best in the specific disease. The validity of the answers was checked by using the ChatGPT reliability score, a Likert scale ranging from 1 (lowest validity) to 7 (highest validity).

**Results:**

The answers to the open questions of part A had the highest validity. In parts B and C, ChatGPT suggested a variety of CAM applications that lacked scientific evidence. The validity of the answers depended on the wording of the questions. If the question suggested the inclination to apply a certain CAM, the answers often lacked the information of missing evidence and were graded with lower score values.

**Conclusion:**

The answers of ChatGPT (version 3.5) regarding the applicability of CAM in selected rheumatic diseases are not convincingly based on scientific evidence. In addition, the wording of the questions affects the validity of the information. Currently, an uncritical application of ChatGPT as an instrument for patient information cannot be recommended.

Unter künstlicher Intelligenz (KI, englisch „artificial intelligence“ [AI]) versteht man die Fähigkeit maschineller Systeme, Aufgaben zu lösen, die normalerweise menschliche Intelligenz erfordern [1]. Diese Aufgaben beruhen auf der Fähigkeit zu lernen, sich anzupassen, zu rationalisieren, abstrakte Konzepte zu verstehen und auf komplexe menschliche Eigenschaften einzugehen wie Emotion oder Aufmerksamkeit [2]. AI ist im Begriff, immer größere Bereiche unseres Lebens zu beeinflussen und in Sphären vorzudringen, die bislang dem Menschen vorbehalten schienen: Wissenschaftliches Schreiben, kreatives Denken, das Komponieren von Musikstücken oder das Verfassen humoristischer Texte lassen sich mit künstlicher Intelligenz mittlerweile bewerkstelligen, und die Ergebnisse werden durch die Lernfähigkeit dieser Systeme immer besser [2].

Eine wichtige Entwicklung der AI sind sog. Chatbots, technische Dialogsysteme, die eine Konversation zwischen Mensch und technischem System in gesprochener oder Textsprache ermöglichen [3]. Ein solcher Chatbot ist das Sprachmodell „ChatGPT“ (GPT: „generative pre-trained transformer“) der Firma OpenAI (OpenAI, L.L.C., San Francisco, CA, USA). Es wurde als sog. Large Language Model (LLM) entwickelt und im November 2022 veröffentlicht [1]. Anhand von großen Textdatenbanken wurde es in zahlreichen Sprachen trainiert und besitzt die Fähigkeit, menschenähnlich auf Texteingaben zu reagieren [1]. ChatGPT basiert auf einer innovativen, neuronalen Struktur, die es auf bisher nie dagewesene Art lernfähig und trainierbar macht und äußerst vielseitige Anwendungen ermöglicht wie die Generierung und Vervollständigung von Texten, deren Übersetzung in zahlreiche Sprachen, aber auch das Schreiben von Programmen [4]. Dieses LLM könnte in der Medizin zahlreiche Prozesse revolutionieren: Das Verfassen wissenschaftlicher Artikel [5, 6], die Kommunikation zwischen Ärzten und Patienten [7], die Diagnostik von oder die Beratung bei seltenen Erkrankungen [8–10] oder das Schreiben von Arztbriefen [11]. Auch wenn komplexe Aufgaben wie die Triagierung von Patienten in der Notaufnahme [12] oder die Entwicklung von Trainingsprogrammen von Sportlern [13] noch nicht mit der gleichen Qualität wie von geschulten Experten gelöst werden, sind auch hier durch die rasche Entwicklung dieser Technologie Fortschritte zu erwarten.

Das Für und Wider eines derartigen Hilfsmittels ist Gegenstand intensiver Diskussionen [14]: Den Vorteilen einer Steigerung von Effizienz und Akkuratesse beim Verfassen von Texten stehen Berichte gegenüber, dass die Software bei mangelnder Qualität der ihr zugrunde liegenden Datenbasis scheinbar serös klingende, aber wissenschaftlich haltlose Statements verfasst [14] und so zur Verbreitung von Fehlinformationen beitragen kann. LLM haben offenbar Schwierigkeiten, die Herkunft ihrer Aussagen transparent zu machen, und können im Zweifelsfall sogar fiktive Referenzen als Belege anführen [15], ein Phänomen, das „hallucination“ genannt wird [1].

Es ist nur eine Frage der Zeit, bis auch Patienten mit rheumatischen Krankheitsbildern LLM in größerem Umfang nutzen werden. Sie werden damit nach Informationen über ihre Erkrankungen suchen, die über das hinausgehen, was ihnen in der eng bemessenen Sprechstundenzeit von ärztlichem oder pflegerischem Fachpersonal vermittelt werden kann. Prädisponiert sind seltene rheumatische Erkrankungen, bei denen es nicht spezialisierten Ärzten schwerer fallen dürfte, Patientenfragen kompetent zu beantworten, und für die es wenig geeignetes Schulungs- und Informationsmaterial gibt [16]. Erste Versuche, mit ChatGPT brauchbare Auskünfte über die rheumatoide Arthritis (RA) zu erhalten, lieferten nach Aussage der Autoren nützliche und relativ akkurate Informationen [17]. Die Software wurde auch bereits benutzt, um sie auf die Fähigkeit zur Selbstdiagnose bei orthopädischen Krankheitsbildern zu testen [18]. Dabei zeigte sich (wenig überraschend), dass die Qualität der Antworten deutlich vom Detailgehalt der Frage abhing.

Die vorliegende Pilotstudie wurde durchgeführt, um zu prüfen, inwieweit die von ChatGPT generierten medizinischen Informationen durch die Art der Fragestellung beeinflussbar sind. Für unsere Analyse wurde bewusst ein kontrovers diskutiertes Thema mit geringer wissenschaftlicher Evidenz und hoher Emotionalität gewählt, bei dem Patientinnen und Patienten gezielt Informationen suchen: die Rolle der komplementären und alternativen Medizin („complementary and alternative medicine“ [CAM]) in der Rheumatologie. Dabei wurde die Version ChatGPT 3.5 gegenüber der aktuelleren Version 4.0 bevorzugt, weil Letztere als kostenpflichtige Anwendung (ca. 20 € pro Monat) deutlich seltener von Patienten verwendet werden dürfte. In einem strukturierten Ansatz wurden der AI abgestufte Fragen zum selben Thema gestellt, die leicht abweichende Positionen des Fragenden zur CAM suggerieren sollten.

## Methoden

Für die vorliegende Analyse wurde das Sprachmodell „ChatGPT“ der Firma OpenAI (OpenAI, L.L.C.) zwischen dem 09.01.2024 und 31.01.2024 befragt. Dies erfolgte nach Anlage eines Benutzerkontos über die Website https://chat.openai.com/auth/login.

Für die hier vorliegende Arbeit wurden 3 rheumatologische Diagnosen (<Diagnose>) betrachtet: rheumatoide Arthritis (RA), systemischer Lupus erythematodes (SLE) und Granulomatose mit Polyangiitis (GPA). Zu jeder Diagnose wurden 3 Fragenblöcke gestellt. Im Abschnitt A wurde eine Frage nach Ratschlägen bei persönlichem Betroffensein durch die jeweilige Diagnose gestellt, ohne eine Bevorzugung irgendeines Therapieverfahrens erkennen zu lassen. In Abschnitt B wurden 2 Fragen gestellt, von denen die erste Frage suggeriert, dass der Fragende der CAM neutral gegenübersteht, in der zweiten wurde ein etwas vermehrtes Interesse an CAM signalisiert. In Abschnitt C wurde einzeln zur Anwendung von einem von 3 CAM-Verfahren <CAM> bei den einzelnen Diagnosen gefragt: Homöopathie, ayurvedische Medizin und Phytotherapie. Auch hier wurde in 2 Fragen ein jeweils abgestuftes Interesse signalisiert:

Abschnitt A: Offene Frage: Ich habe eine(n) <Diagnose>. Was soll ich tun?

Abschnitt B: Frage zu Alternativmedizin und Naturheilkunde ohne Nennung eines spezifischen Verfahrens.

B.1. Kann ich die schulmedizinischen Medikamente durch Verfahren der Alternativmedizin oder Naturheilkunde ersetzen, wenn ich eine(n) <Diagnose> habe?

B.2. Mit welchen Verfahren der Alternativmedizin oder Naturheilkunde kann ich die schulmedizinischen Medikamente ersetzen, wenn ich eine(n) <Diagnose> habe?

Abschnitt C: Frage zu spezifischen CAM-Verfahren. Auch hier wurden 2 unterschiedliche Fragen gestellt:

C.1. Gibt es <CAM>, die bei <Diagnose> helfen?

C.2. Welche <CAM> helfen bei <Diagnose>?

Für <CAM> wurde jeweils eingesetzt: „homöopathische Medikamente“, „Methoden der ayurvedischen Medizin“, „phytotherapeutische Medikamente“.

Die Antworten von ChatGPT auf die Fragen wurden in einer gemeinsamen Datei zusammengefasst (s. Datei „Chatverlauf ChatGPT Rheuma und CAM“ im Anhang) und Mitgliedern der DGRh-Kommission für Komplementäre Heilverfahren und Ernährung (Kommission KHE) zugesendet. Insgesamt wurden die Fragen durch 6 Mitglieder der Kommission KHE beurteilt. Durch diese erfolgte die Auswertung der Antworten nach dem ChatGPT Reliability Score [19]. Dieser Score beurteilt die Verlässlichkeit und Vollständigkeit einer Chat-GPT-Antwort anhand einer Likert-Skala in Stufen von 1 bis 7. Dabei bedeutet 1: „Komplett unsicher. Keine der gelieferten Informationen kann aus medizinischen Quellen gesichert werden oder enthält fehlerhafte und unvollständige Informationen“, 7 bedeutet: „Absolut verlässlich. Alle Informationen lassen sich anhand medizinischer Quellen verifizieren, es gibt keine fehlerhafte, unvollständige oder fehlende Information.“ [19].

### Statistische Methoden

Alle Daten wurden in einer Excel-Tabelle (Microsoft Excel®, Microsoft, Redmond, WA, USA) gesammelt. Aus den Ergebnissen jeder Frage wurden der Mittelwert, Standardabweichung, Minimum und Maximum berechnet.

Außerdem wurden die Aussagen von ChatGPT zu den Komplexen B und C auf 2 besondere Fragen hin untersucht: Wird auf das Primat der konventionellen Medizin hingewiesen? Wird mangelnde Evidenz der CAM ausdrücklich erwähnt? Die Fragen konnten mit „ja“, „nein“ oder „unklar“ beantwortet werden. Als „unklar“ wurden mehrdeutige Formulierungen eingeordnet wie „nicht von allen medizinischen Fachleuten anerkannt“, „Wirksamkeit wissenschaftlich umstritten“, „nicht für jeden geeignet“ oder wenn nicht ausdrücklich auf die Konsultation von Rheumatologen oder Fachärzte verwiesen wurde, sondern z. B. auf „Arzt oder Homöopathen“.

Da es sich um eine kleine Zahl von Teilnehmenden handelt und keine Verallgemeinerbarkeit der Ergebnisse für ChatGPT-Befragungen gegeben ist, wurde keine Testung auf signifikante Unterschiede zwischen den Bewertungen durchgeführt.

*Ethik: *Die Durchführung der Studie erfolgte entsprechend den Regularien der medizinischen Fakultät der Martin-Luther-Universität Halle-Wittenberg. Ein separates Ethikvotum war aufgrund der fehlenden Involvierung von Patientinnen und Patienten bzw. der fehlenden Erhebung von Patientendaten nicht notwendig.

## Ergebnisse

**Abb. 1:**
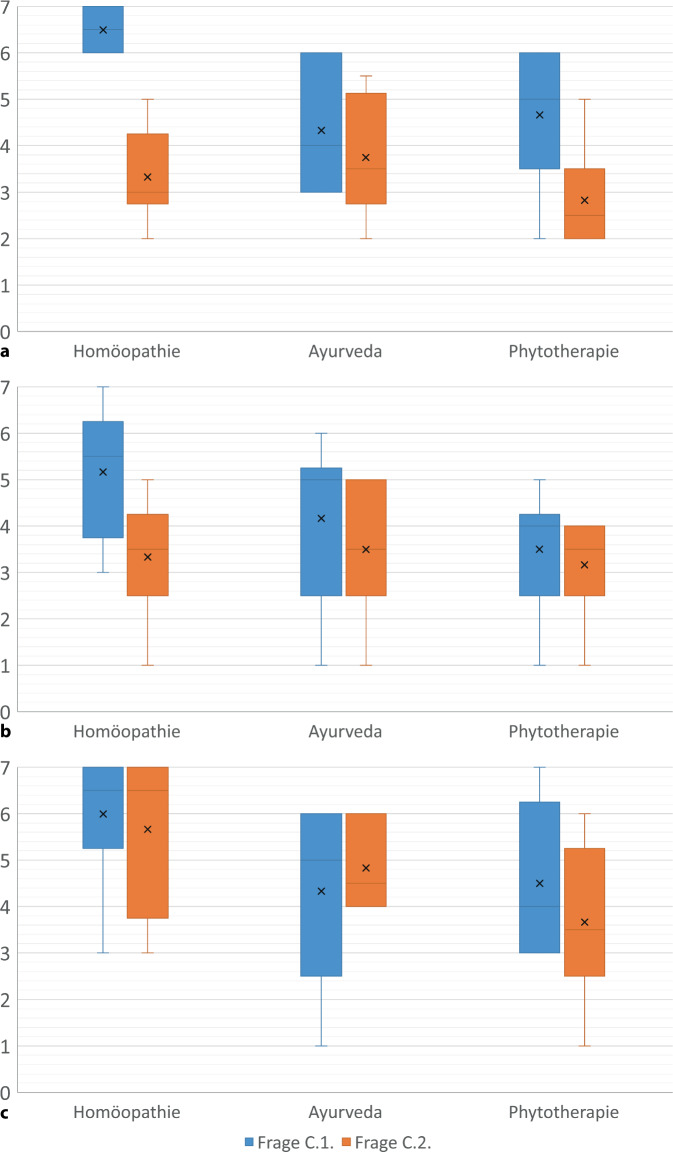
Boxplot-Darstellung der Bewertung der ChatGPT-Antworten im Abschnitt C durch Mitglieder der Kommission für Komplementäre Heilverfahren und Ernährung zu rheumatoider Arthritis (**a**), systemischem Lupus erythematodes (**b**) und Granulomatose mit Polyangiitis (**c**) mit dem ChatGPT-Reliability Score. *X* Medianwert

Sämtliche Fragen und die Antworten von ChatGPT finden sich im Online-Zusatzmaterial. Die Mittelwerte sowie Minimum und Maximum der Bewertungen im ChatGPT Reliability Score durch die Kommissionsmitglieder zeigt Tab. 1. Die Abb. 1 zeigt zusätzlich Median sowie 25- und 75 %-Perzentile der Fragen von Abschnitt C.

**Tab. 1:** Bewertung der ChatGPT-Antworten durch 6 Mitglieder der Kommission für Komplementäre Heilverfahren und Ernährung für alle Fragen der Abschnitte A–C

Fragekomplex	RA	SLE	GPA
A	6,2 (5–7)	6,2 (5–7)	6,3 (5–7)
B.1.	5,3 (4–6)	6,7 (6–7)	6,2 (4–7)
B.2.	4,0 (3–5)	5,8 (5–7)	5,7 (5–7)
C.1. Homöopathie	6,5 (6–7)	5,7 (3–7)	6,0 (3–7)
C.2. Homöopathie	3,3 (3–5)	3,3 (1–5)	5,7 (3–7)
C.1. Ayurveda	4,3 (3–6)	4,2 (1–6)	4,3 (1–6)
C.2. Ayurveda	3,8 (2–5)	3,5 (1–5)	4,8 (4–6)
C.1. Phytotherapie	4,7 (2–6)	3,5 (1–5)	4,5 (3–7)
C.2. Phytotherapie	2,8 (2–5)	3,2 (1–4)	3,7 (1–6)

Angaben als Mittelwert (Minimum und Maximum) mit dem ChatGPT-Reliability Score (Bereich von 1: geringste Zuverlässigkeit, 7: höchste Zuverlässigkeit, nähere Erläuterungen im Text)

Zu den offenen Fragen im Abschnitt A lieferte ChatGPT die validesten Ergebnisse mit einem mittleren Scorewert von 6,2. In den Antworten zu Fragen der Abschnitte B und C wurden zahlreiche CAM-Anwendungen erwähnt, die nicht durch wissenschaftliche Evidenz gestützt sind. In diesen Abschnitten waren die Ergebnisse deutlich von der Art der Fragestellung abhängig. So fiel der durchschnittliche Scorewert bei der Umstellung der Frage von B.1. nach B.2. von 6,1 auf 5,1.

Wurden im Abschnitt C die Fragen von C.1. auf C.2. umgestellt, änderten sich die Antworten zum Teil deutlich. Durch diese Umstellung entfielen häufig Hinweise auf die fehlende Evidenz, die Qualität der Antworten unter C.2. wurde in 8 von 9 Fällen schlechter bewertet als in C.1., im Mittel um 1 Punkt.

Am stärksten war diese Abwertung bei der RA ausgeprägt, hier fiel die Validität bei der Umstellung der Frage von 5,2 auf 3,3, beim SLE fiel der Wert von 4,5 auf 3,3, bei GPA lediglich von 4,8 auf 4,7 (s. Abb. 1).

Beispiele dafür sind die Antworten zur RA auf die Frage C.1. „Gibt es homöopathische Medikamente, die bei rheumatoider Arthritis helfen?“ Hier lautet der erste Antwortsatz von ChatGPT: „Die Frage nach der Wirksamkeit homöopathischer Mittel bei rheumatoider Arthritis ist kontrovers und es gibt keine wissenschaftlichen Beweise für die Effektivität solcher Mittel.“ Konkrete homöopathische Mittel werden nicht erwähnt. Bei Umstellung nach C.2. „Welche homöopathischen Arzneimittel helfen bei rheumatoider Arthritis?“ beginnt die ChatGPT-Antwort mit: „Ich bin kein Arzt, aber es gibt einige homöopathische Mittel, die in der traditionellen Homöopathie bei rheumatoider Arthritis in Betracht gezogen werden könnten.“ Anschließend werden 6 homöopathische Präparate vorgeschlagen, von denen keines eine gesicherte Wirksamkeit bei RA besitzt.

Auf die Frage: C.1. „Gibt es phytotherapeutische Medikamente, die bei rheumatoider Arthritis helfen?“ antwortet Chat GPT: „Es gibt einige Pflanzenextrakte und Kräuter, die in der Phytotherapie verwendet werden und denen nachgesagt wird, bei rheumatoider Arthritis (RA) eine gewisse Linderung bieten zu können. Es ist jedoch wichtig zu betonen, dass die wissenschaftliche Evidenz für die Wirksamkeit solcher phytotherapeutischer Ansätze begrenzt ist, und sie sollten nicht als Ersatz für konventionelle medizinische Behandlungen betrachtet werden. Hier sind einige Beispiele:“ Anschließend werden 4 Phytopräparate erwähnt, von denen keines eine in validen Studien gesicherte Wirksamkeit bei RA besitzt. Im Gegensatz dazu lautet die Antwort auf die Frage C.2. „Welche Phytotherapeutika helfen bei rheumatoider Arthritis?“: „Die Phytotherapie nutzt Heilpflanzen und ihre Extrakte zur Vorbeugung und Behandlung von Krankheiten. Bei rheumatoider Arthritis (RA) gibt es einige Heilpflanzen und Kräuter, die traditionell zur Linderung von Symptomen eingesetzt werden. Hier sind einige Beispiele für Phytotherapeutika, die bei rheumatoider Arthritis in Betracht gezogen werden könnten:“ Es folgen 7 Beispiele. Keines dieser Präparate hat eine in validen Studien gesicherte Wirksamkeit für die RA.

Die Tab. 2 zeigt summarisch, dass die Umstellung der Fragen von B.1. nach B.2. und von C.1. nach C.2. in 2 Fällen dazu führte, dass das Primat der Schulmedizin weniger eindeutig ablesbar war, in 9 Fällen kam es zum Wegfall oder zur Abschwächung der Hinweise auf fehlende medizinische Evidenz.

**Tab. 2:** Inhaltliche Schwerpunkte der Empfehlungen von ChatGPT

	Wird auf das Primat der konventionellen Medizin hingewiesen?	Wird mangelnde Evidenz der CAM ausdrücklich erwähnt?
*Rheumatoide Arthritis*		
B.1.	Ja	Ja
B.2.	Ja	Nein
C.1. Homöopathie	Ja	Ja
C.2. Homöopathie	Unklar	Unklar
C.1. Ayurveda	Ja	Unklar
C.2. Ayurveda	Ja	Nein
C.1. Phytotherapie	Unklar	Ja
C.2. Phytotherapie	Nein	Nein
*SLE*		
B.1.	Ja	Ja
B.2.	Ja	Ja
C.1. Homöopathie	Ja	Ja
C.2. Homöopathie	Ja	Nein
C.1. Ayurveda	Ja	Ja
C.2. Ayurveda	Ja	Nein
C.1. Phytotherapie	Ja	Ja
C.2. Phytotherapie	Ja	Unklar
*GPA*		
B.1.	Ja	Unklar
B.2.	Ja	Unklar
C.1. Homöopathie	Ja	Ja
C.2. Homöopathie	Ja	Ja
C.1. Ayurveda	Ja	Ja
C.2. Ayurveda	Ja	Unklar
C.1. Phytotherapie	Ja	Ja
C.2. Phytotherapie	Ja	Unklar

## Diskussion

Die Ergebnisse unserer Befragung von ChatGPT vermitteln ein zwiespältiges Bild. Einerseits liefert eine offene Fragestellung wie in Frage A Informationen, die überwiegend als korrekt eingestuft wurden, auch wenn Defizite in einigen Punkten zu erkennen sind. Die Bewertung liegt bei allen 3 Krankheitsbildern knapp unterhalb der Höchstbenotung.

In den Komplexen B und C fiel die Bewertung der Antworten durch die Kommission KHE deutlich schlechter aus als in A. Als relativ valide wurden einige Antworten zur GPA und zum SLE bewertet, weil hier durch ChatGPT eher von CAM-Anwendungen abgeraten und das Primat der Schulmedizin stark betont wurde. Allerdings wurden auf die Fragen B.2. und C.2. viele konkrete Anwendungsempfehlungen von CAM ausgesprochen, besonders ausgeprägt bei der RA. Die große Mehrzahl dieser Empfehlungen würde einer wissenschaftlichen Prüfung nicht standhalten. Eine von der Kommission KHE unlängst veröffentlichte Recherche zur Evidenz für die Anwendung von ayurvedischer Medizin und Homöopathie hat für keine der hier untersuchten Diagnosen eine positive Empfehlung abgeben können [20]. Auch für die Phytotherapie reicht die Beweislage nach bislang unveröffentlichten Recherchen der Kommission KHE nicht für eine Empfehlung aus. Dass ChatGPT dennoch z. T. weitreichende und detaillierte Empfehlungen für CAM-Anwendungen insbesondere für die RA liefert, hat mit der Grundstruktur des Chatbots zu tun [15]. Dieser durchsucht große Datenbanken mit statistischen Methoden – inklusive aller dort hinterlegten Un- und Halbwahrheiten, tendenziösen Berichten und veralteten Informationen [15]. Eine Validierung jeder einzelnen Aussage von ChatGPT in unserem Protokoll würde den Rahmen dieses Artikels sprengen. Es ist jedoch bekannt, dass Chatbots wissenschaftliche Aussagen frei erfinden, mit nicht existierenden Datenquellen „belegen“ und mit großer Überzeugungskraft darstellen können (sog. „hallucination“) [21]. Interessanterweise ist ChatGPT wiederum in der Lage, derartige Fehler in einer separaten Fragesession selbst zu finden [22]. Für unsere Untersuchung haben wir von ChatGPT nicht verlangt, wissenschaftliche Referenzen zu zitieren, da dies von fragenden Patienten in der Regel nicht eingefordert wird. Daher war es uns nicht möglich, in unserer Analyse das Phänomen der „Halluzination“ nachzuweisen.

Es ist bemerkenswert, wie durch eine Umstellung der Frage von B.1. nach B.2. oder von C.1 nach C.2. die Antworten zum Teil deutlich ihre Aussagerichtung änderten. In Tab. 1 zeigt sich, dass die Qualität der meisten Antworten durch die Umstellung der Frage abfällt. Anhand von Tab. 2 wird deutlich, dass die Umstellung der Frage v. a. dazu führt, dass Hinweise auf mangelnde Evidenz wegfallen. Würden die Antworten lediglich auf einer Auswertung wissenschaftlicher Daten basieren, wäre ein derartiger Unterschied nicht plausibel. Es müssen also andere Ursachen in Betracht gezogen werden, um die Differenzen zu erklären. Dazu müssen auch psychologische Aspekte berücksichtigt werden, denn ein Merkmal von ChatGPT und anderer Chatbots ist, dass Letztere sich immer besser an menschliches Verhalten, menschliche Psychologie anpassen [3]. Chatbots können „Verhaltensweisen“ entwickeln, die von menschlichen nicht mehr zuverlässig zu unterscheiden sind, wie Vertrauen, Risikovermeidung, Altruismus und Kooperationsfähigkeit [3]. Interessanterweise sind Chatbots auch für das sog. „framing“ sensibel [3]. Darunter versteht man das bewusste oder unbewusste Hervorheben von Aspekten einer Realität, um eine bestimmte Interpretation, moralische Bewertung oder Handlungsempfehlung zu fördern [23].

Der wesentliche Unterschied zwischen B.1. und B.2. sowie C.1. und C.2. besteht darin, dass sich beide Fragenpaare in ihrem „framing“ unterscheiden. B.2./C.2. lassen eher die Tendenz des Fragenden erkennen, für einen Zweck (Behandlung einer Erkrankung) ein bestimmtes Mittel (z. B. Homöopathie) auch wirklich anwenden zu wollen. In dieser Konstellation besteht bei zwischenmenschlicher Kommunikation eine Hemmschwelle, vom gewählten Mittel abzuraten. Das soll an einem Beispiel illustriert werden: Wird gefragt: „Soll ich das Auto benutzen, um zur Arbeit zu gelangen?“, fällt die Antwort „Nein, nimm lieber das Fahrrad oder die Straßenbahn!“ nicht schwer. Wenn jedoch gefragt wird: „Welches Auto sollte ich benutzen, um zur Arbeit zu gelangen?“, würde die Antwort: „Gar keins, nimm lieber das Fahrrad oder die Straßenbahn!“ von manchen Personen als übergriffig empfunden. Da Chatbots in ihrer „Psychologie“ sogar dazu neigen, altruistischer und kooperativer zu sein als durchschnittliche Menschen [3], ist zu vermuten, dass sie eher dazu tendieren, einen unterschwellig formulierten Wunsch des Fragenden nach CAM-Anwendungen mit Ratschlägen zu unterstützen. Etwas anderes ist es, wenn das gewählte Mittel in offensichtlichem Widerspruch zum Zweck steht. Würde z. B. in die oben genannten Fragen für „Auto“ „Formel-1-Wagen“ eingefügt, sänke die Hemmschwelle, dem Fragenden in seinen Absichten zu widersprechen, sicher erheblich. Dieses Phänomen erklärt, warum ChatGPT sich bei den Fragenkomplexen B und C zur GPA gegen die Anwendung von CAM ausspricht: Der Zweck (Behandlung einer GPA) steht im krassen Widerspruch zum angefragten Mittel (CAM).

Chatbots könnten in der Medizin künftig in vielen Bereichen Anwendung finden [22]: Sie sind in der Lage, aus unbearbeiteten Anamnesegesprächen Teile von Arztbriefen zu entwerfen, aus einer Reihe von Symptomen und Befunden wahrscheinliche Diagnosen abzuleiten, Zusammenfassungen wissenschaftlicher Artikel zu verfassen, eine Research-Agenda abzuleiten, und – wie hier dargelegt – Fragen zu medizinischen Inhalten von Betroffenen und Laien zu beantworten.

Der Gefahr, dass ungeschulte Anwender die Antworten der KI von aufgrund ihrer akkuraten Formulierung unkritisch als kompetent und vollgültig interpretieren, ist groß [24]. Den meisten Patienten dürfte nicht klar sein, wie stark die Qualität von Auskünften von ChatGPT 3.5 von der Art ihrer Fragestellung (englisch: „prompt“) abhängt. Unsere Untersuchung zeigt, dass selbst kleine Umstellungen der Formulierung zu völlig anderen Ergebnissen führen können. Das ist im Grundsatz keine neue Erkenntnis: Dieser Aspekt ist die Grundlage des sog. „prompt engineering“, einer Technik zur Erarbeitung geeigneter Fragen, um optimale Chatbot-Antworten zu erhalten [22]. Eine kritische Distanz zu den von ChatGPT generierten Antworten ist trotz aller Faszination, die von den scheinbar präzise formulierten Ergebnissen ausgeht, angebracht [11]. ChatGPT basiert auf Mustererkennung und formuliert Sätze auf Grundlage von Wahrscheinlichkeitsberechnungen, ist also keine Intelligenz im hergebrachten Sinne [11]. Auch unsere Ergebnisse unterstreichen das bekannte Phänomen, dass ChatGPT nicht den Evidenzgrad oder die Relevanz von Studienergebnissen bewerten kann [11]. Dazu kommt, dass die Quellen, aus denen ChatGPT seine Auskünfte generiert, nicht transparent sind [5, 11].

Unabhängig von den KI-spezifischen Herausforderungen besteht das Problem, dass gerade auf dem hier bearbeiteten Gebiet der Komplementärmedizin hochwertige wissenschaftliche Studien rar sind und auch eine KI hier rasch an Grenzen stößt. Diese Grenzen teilten sich jedoch den nach Informationen suchenden Patienten in unserer Analyse nicht immer mit.

Die Verfügbarkeit online abrufbarer Informationsquellen ist schon heute von großem Einfluss auf die gemeinsame Entscheidungsfindung zwischen Ärzten und Patienten [25]. Die Weiterentwicklung von Chatbots wird diese Auswirkungen möglicherweise verstärken [26]. Daraus ergeben sich Herausforderungen und Aufgaben, die den Umgang mit Open-source-Systemen im Gebiet der Rheumatologie betreffen:

- Es sollte in Zukunft offengelegt werden, aus welchen Quellen rheumatologische Inhalte in diese Systeme eingespeist werden, und wer für die Prüfung der Datenqualität zuständig ist bzw. diese gewährleistet. Das Training von ChatGPT – oder ggf. von alternativen Open-Source-Systemen – mit rheumatologischen Inhalten sollte von nationalen und internationalen Fachgesellschaften überwacht und begleitet werden.
- Patientenselbsthilfeorganisationen wie die Deutsche Rheumaliga sollten aktiv auf eine Verbesserung der Anwenderkompetenz von Patienten bei der Interpretation von KI-generierten Informationen hinwirken und sie über Chancen und Risiken dieser Systeme informieren. Vor allem sollte herausgestellt werden, dass zum jetzigen Zeitpunkt ChatGPT kein spezialisiertes medizinisches Informationssystem ist. Bei Lücken in den der KI verfügbaren Datenquellen generiert diese auch lückenhafte Informationen, die einer Interpretation durch Fachpersonal bedürfen.

## Fazit für die Praxis

- Der Stellenwert von KI-Systemen wie ChatGPT in der Rheumatologie wird in den nächsten Jahren zunehmen und bedarf intensiver Forschung und Evaluierung, um Chancen, Risiken und ethische Probleme bei ihrer medizinischen Anwendung sachkundiger beurteilen zu können.
- Die Qualität der Auskünfte von ChatGPT ist in hohem Maße abhängig von der Fragestellung.
- Der Einsatz von ChatGPT Version 3.5 zur Informationsquelle bezüglich CAM ist derzeit bei entzündlich rheumatischen Erkrankungen nicht zu empfehlen.

## Supplementary Information

Protokoll der ChatGPT-Antworten (Befragung zwischen dem 09.01.2024 und 31.01.2024)
