# Supplementary material for: What is the potential of ChatGPT for qualified patient information? Attempt of a structured analysis on the basis of a survey regarding complementary and alternative medicine (CAM) in rheumatology
Source: Z Rheumatol. 2024 Jul 10;84(3):179–87. [Article in German] doi: 10.1007/s00393-024-01535-6 (PMC11965147; doi:10.1007/s00393-024-01535-6)
Supplement: Supplementary file 1 — Protokoll der ChatGPT-Antworten (Befragung zwischen dem 09.01.2024 und 31.01.2024) [file 393_2024_1535_MOESM1_ESM.pdf]

**Welches Potential hat ChatGPT 3.5 für eine qualifizierte Patienteninformation? Versuch einer systematischen Analyse anhand einer Befragung zu komplementärmedizinischen Verfahren in der Rheumatologie**

**Protokoll der Chat-GPT-Antworten (Befragung zwischen dem 9.1.2024 und 31.1.2024)**

**Rheumatoide Arthritis (RA)**

**A. Ich habe eine rheumatoide Arthritis. Was soll ich tun?**

Es tut mir leid zu hören, dass Sie rheumatoide Arthritis haben. Es ist wichtig, einen Rheumatologen oder einen anderen Facharzt aufzusuchen, um eine genaue Diagnose zu erhalten und einen Behandlungsplan zu entwickeln. Hier sind einige allgemeine Empfehlungen und Schritte, die Sie in Betracht ziehen können:

1. **Medizinische Beratung**: Konsultieren Sie einen Rheumatologen oder einen anderen Facharzt für Rheumatologie. Ein Rheumatologe ist spezialisiert auf die Diagnose und Behandlung von Erkrankungen des Bewegungsapparates und des Bindegewebes, einschließlich rheumatoider Arthritis.

2. **Behandlungsplan**: Ihr Arzt wird Ihnen wahrscheinlich eine Kombination aus Medikamenten und anderen Therapieansätzen empfehlen. Dies kann entzündungshemmende Medikamente, Immunmodulatoren, Physiotherapie und möglicherweise auch chirurgische Eingriffe umfassen.

3. **Medikamentöse Therapie**: Verschiedene Medikamente können zur Behandlung von rheumatoider Arthritis eingesetzt werden, darunter nichtsteroidale entzündungshemmende Medikamente (NSAIDs), Steroide, Methotrexat und Biologika.

4. **Lebensstil und Selbstmanagement**: Überlegen Sie, wie Sie Ihren Lebensstil anpassen können, um Symptome zu lindern und die Lebensqualität zu verbessern. Dies kann eine ausgewogene Ernährung, regelmäßige Bewegung, Stressmanagement und genügend Ruhe beinhalten.

5. **Unterstützungsgruppen**: Es kann hilfreich sein, sich einer Selbsthilfegruppe für Menschen mit rheumatoider Arthritis anzuschließen. Der Austausch mit anderen Betroffenen kann emotionalen Beistand bieten und praktische Tipps für den Umgang mit der Erkrankung liefern.

6. **Regelmäßige Kontrolluntersuchungen**: Bleiben Sie in engem Kontakt mit Ihrem Arzt und nehmen Sie regelmäßige Kontrolluntersuchungen wahr, um den Verlauf Ihrer Erkrankung zu überwachen und den Behandlungsplan bei Bedarf anzupassen.

7. **Bildung und Informationsbeschaffung**: Informieren Sie sich über die Erkrankung, um ein besseres Verständnis für Ihre Symptome, den Krankheitsverlauf und die verfügbaren Behandlungsoptionen zu erhalten.

Denken Sie daran, dass jeder Mensch individuell ist und dass der beste Behandlungsansatz von vielen Faktoren abhängt, einschließlich des Schweregrads der Erkrankung, Ihrer allgemeinen Gesundheit und Ihrer persönlichen Vorlieben. Es ist wichtig, einen umfassenden Ansatz zu wählen und eng mit Ihrem medizinischen Team zusammenzuarbeiten, um die bestmögliche Versorgung zu gewährleisten.

**B.1. Kann ich die schulmedizinischen Medikamente durch Verfahren der Alternativmedizin oder Naturheilkunde ersetzen, wenn ich eine rheumatoide Arthritis habe?**

Ich bin kein Arzt, aber ich kann Ihnen einige Informationen bereitstellen, die Ihnen helfen können, eine informierte Entscheidung zu treffen.

Rheumatoide Arthritis (RA) ist eine chronische Autoimmunerkrankung, die die Gelenke betrifft. Die schulmedizinische Behandlung von RA konzentriert sich in der Regel auf die Linderung von Symptomen und die Verlangsamung des Fortschreitens der Erkrankung. Dazu gehören oft nichtsteroidale Antirheumatika (NSAR), Corticosteroide und sogenannte Disease-Modifying Antirheumatic Drugs (DMARDs).

Einige Menschen mit RA suchen auch nach alternativen oder ergänzenden Therapien, um ihre Symptome zu lindern oder die Wirkung schulmedizinischer Behandlungen zu unterstützen. Einige gängige Ansätze in der Alternativmedizin und Naturheilkunde bei RA können sein:

1. **Akupunktur**: Einige Studien haben gezeigt, dass Akupunktur Schmerzen bei RA lindern kann, obwohl die Ergebnisse gemischt sind.

2. **Kräutermedizin**: Einige Kräuter wie Weihrauch (Boswellia serrata) und Kurkuma (Curcumin) werden manchmal zur Behandlung von RA eingesetzt. Es ist wichtig, mit einem Arzt zu sprechen, bevor Sie Kräuterpräparate einnehmen, da sie Nebenwirkungen haben können und mit anderen Medikamenten interagieren können.

3. **Omega-3-Fettsäuren**: Diese können entzündungshemmende Eigenschaften haben und werden manchmal zur Behandlung von RA eingesetzt.

4. **Ernährung**: Einige Menschen mit RA berichten von einer Linderung ihrer Symptome durch bestimmte Ernährungsumstellungen, wie z.B. eine glutenfreie oder entzündungshemmende Ernährung.

Es ist wichtig zu beachten, dass die wissenschaftliche Evidenz für viele dieser alternativen Ansätze begrenzt ist. Bevor Sie irgendwelche Änderungen an Ihrer Behandlung vornehmen oder alternative Therapien ausprobieren, sollten Sie unbedingt mit Ihrem Arzt oder einem Rheumatologen sprechen. Es ist auch wichtig, dass Sie nicht aufhören, Ihre verschriebenen Medikamente ohne Anweisung Ihres Arztes einzunehmen, da dies zu einem Schub der Krankheit oder anderen gesundheitlichen Problemen führen kann.

**B.2. Mit welchen Verfahren der Alternativmedizin oder Naturheilkunde kann ich die schulmedizinischen Medikamente ersetzen, wenn ich eine rheumatoide Arthritis habe?**

Ich bin kein Arzt, aber viele Menschen mit rheumatoider Arthritis suchen nach alternativen oder ergänzenden Therapien zur Linderung ihrer Symptome. Es ist wichtig, dass Sie bei einer chronischen Erkrankung wie rheumatoider Arthritis immer einen qualifizierten Mediziner oder Rheumatologen konsultieren, bevor Sie Änderungen an Ihrer Behandlung vornehmen oder alternative Therapien ausprobieren.

Einige Ansätze aus der Alternativmedizin und Naturheilkunde, die manchmal bei rheumatoider Arthritis erwogen werden, sind:

1. **Akupunktur und Akupressur**: Einige Studien haben gezeigt, dass Akupunktur die Schmerzen bei rheumatoider Arthritis lindern kann. Akupressur verwendet Druckpunkte ohne Nadeln.

2. **Omega-3-Fettsäuren**: Fischöl, das reich an Omega-3-Fettsäuren ist, kann entzündungshemmende Eigenschaften haben und die Symptome lindern.

3. **Ingwer**: Einige Studien haben gezeigt, dass Ingwer entzündungshemmende Eigenschaften haben kann und bei einigen Menschen mit rheumatoider Arthritis zu einer Linderung der Symptome führen kann.

4. **Kurkuma und Curcumin**: Kurkuma ist ein Gewürz, das Curcumin enthält, das entzündungshemmende Eigenschaften haben kann. Einige Studien haben vorgeschlagen, dass es bei rheumatoider Arthritis hilfreich sein könnte.

5. **Massage**: Massage kann helfen, die Muskelspannung zu lindern und die Durchblutung zu verbessern, was einige Menschen mit rheumatoider Arthritis als lindernd empfinden.

6. **Entspannungstechniken**: Methoden wie Meditation, progressive Muskelentspannung und tiefe Atemübungen können helfen, den Stress zu reduzieren, der zu einer Verschlimmerung der Symptome beitragen kann.

Es ist wichtig zu betonen, dass diese Ansätze nicht als Ersatz für schulmedizinische Behandlungen angesehen werden sollten, sondern als Ergänzungen. Jeder Mensch reagiert unterschiedlich auf Therapien, und was für eine Person funktioniert, funktioniert möglicherweise nicht für eine andere. Es ist entscheidend, einen umfassenden Behandlungsplan zu haben, der von einem medizinischen Fachmann überwacht wird.

Wenn Sie darüber nachdenken, Ihre Behandlung zu ändern oder alternative Therapien auszuprobieren, sprechen Sie zuerst mit Ihrem Arzt oder Rheumatologen.

**C1. Gibt es homöopathische Medikamente, die bei rheumatoider Arthritis helfen?**

Die Frage nach der Wirksamkeit homöopathischer Mittel bei rheumatoider Arthritis ist kontrovers und es gibt keine wissenschaftlichen Beweise für die Effektivität solcher Mittel. Rheumatoide Arthritis ist eine ernste Erkrankung, die eine angemessene medizinische Behandlung erfordert.

Homöopathie basiert auf dem Prinzip der Selbstheilungskräfte des Körpers und der Verwendung stark verdünnter Substanzen, die ähnliche Symptome wie die Krankheit selbst verursachen. Es gibt jedoch keine überzeugenden wissenschaftlichen Beweise dafür, dass homöopathische Mittel bei rheumatoider Arthritis wirksam sind.

Es ist wichtig zu betonen, dass die Behandlung von rheumatoider Arthritis am besten unter der Anleitung eines qualifizierten medizinischen Fachpersonals erfolgen sollte. Moderne medizinische Ansätze, einschließlich entzündungshemmender Medikamente, krankheitsmodifizierender Antirheumatika (DMARDs) und Biologika, haben sich als wirksam erwiesen, um die Symptome zu lindern und den Krankheitsverlauf zu verlangsamen.

Bevor Sie alternative oder ergänzende Therapieansätze in Erwägung ziehen, ist es wichtig, mit Ihrem Arzt zu sprechen, um sicherzustellen, dass sie sicher und mit den konventionellen Behandlungen kompatibel sind. Selbstmedikation mit homöopathischen Mitteln ohne ärztliche Aufsicht kann zu unerwünschten Ergebnissen führen.

**C.2. Welche homöopathischen Arzneimittel helfen bei rheumatoider Arthritis?**
Ich bin kein Arzt, aber es gibt einige homöopathische Mittel, die in der traditionellen Homöopathie bei rheumatoider Arthritis in Betracht gezogen werden könnten. Homöopathische Behandlungen basieren auf dem Konzept, dass "Ähnliches mit Ähnlichem behandelt werden sollte". Das bedeutet,

dass eine Substanz, die bei gesunden Menschen bestimmte Symptome verursacht, in einer sehr verdünnten Form verwendet wird, um ähnliche Symptome bei Kranken zu behandeln.

Einige homöopathische Mittel, die bei rheumatoider Arthritis in Erwägung gezogen werden könnten, sind:

Rhus toxicodendron: Wird oft für steife und schmerzende Gelenke empfohlen, die sich durch Bewegung verbessern und durch Ruhe verschlechtern.

Bryonia: Für Schmerzen, die durch Bewegung und Berührung verschlimmert werden, aber durch Ruhe gelindert werden.

Apis mellifica: Wenn die Gelenke geschwollen, heiß und stechend sind und sich durch kalte Anwendungen verbessern.

Causticum: Wird für steife Gelenke empfohlen, die durch Kälte und Nässe verschlechtert werden und durch Wärme und Bewegung verbessert werden.

Arnica montana: Wird bei akuten Gelenkschmerzen nach Verletzungen oder Überanstrengung empfohlen.

Belladonna: Für plötzlich auftretende und heftige Schmerzen mit Rötung und Hitze in den betroffenen Gelenken.

Es ist wichtig zu betonen, dass die Homöopathie nicht von allen medizinischen Fachleuten anerkannt wird, und die Wirksamkeit homöopathischer Mittel ist wissenschaftlich umstritten. Vor der Anwendung homöopathischer Mittel bei rheumatoider Arthritis oder einer anderen Erkrankung ist es ratsam, einen Arzt oder Homöopathen zu konsultieren. Es ist auch wichtig, dass Patienten, die bereits eine konventionelle Behandlung für rheumatoide Arthritis erhalten, diese Behandlung nicht ohne Rücksprache mit ihrem Arzt abbrechen oder durch homöopathische Mittel ersetzen.

### C.1. Gibt es Methoden der ayurvedischen Medizin, die bei rheumatoider Arthritis helfen?

Die ayurvedische Medizin, eine traditionelle Heilkunst aus Indien, betrachtet den Körper als Ganzes und strebt eine Harmonie von Geist, Körper und Seele an. Bei rheumatoider Arthritis (RA) gibt es einige ayurvedische Ansätze, die helfen können, die Symptome zu lindern. Es ist jedoch wichtig zu beachten, dass diese Ansätze keine wissenschaftlich nachgewiesene Heilung bieten und dass die Wirksamkeit von Person zu Person variieren kann.

Einige allgemeine ayurvedische Empfehlungen für die Linderung von Rheuma-Symptomen könnten folgendes beinhalten:

1. **Diät:** Ayurveda legt Wert auf eine ausgewogene Ernährung. Empfohlen werden Lebensmittel, die als entzündungshemmend gelten, wie frisches Gemüse, Ingwer, Knoblauch, Kurkuma und bestimmte Kräuter.
2. **Kräuterpräparate:** Einige ayurvedische Kräuterpräparate werden zur Unterstützung bei entzündlichen Erkrankungen, einschließlich rheumatoider Arthritis, verwendet. Zu diesen Kräutern gehören Ashwagandha, Guggul, Triphala, und Kurkuma. Es ist wichtig, diese nur unter Anleitung eines erfahrenen Ayurveda-Praktikers einzunehmen.
3. **Ayurvedische Massagen (Abhyanga):** Spezielle Massagen mit warmem Öl können zur Linderung von Muskelsteifheit und -schmerzen beitragen. Dies sollte jedoch von einem qualifizierten Ayurveda-Therapeuten durchgeführt werden.

4. **Yoga und Meditation:** Ayurveda betont auch die Bedeutung von Yoga und Meditation zur Förderung der Entspannung, Stressreduktion und Verbesserung der Beweglichkeit.

Es ist entscheidend zu beachten, dass ayurvedische Ansätze bei rheumatoider Arthritis nicht als Ersatz für konventionelle medizinische Behandlungen dienen sollten. Moderne medizinische Ansätze, wie entzündungshemmende Medikamente und krankheitsmodifizierende Antirheumatika (DMARDs), sind oft notwendig, um den Krankheitsverlauf zu kontrollieren.

Bevor Sie irgendwelche ayurvedischen Behandlungen in Betracht ziehen, ist es ratsam, dies mit Ihrem behandelnden Arzt zu besprechen, um sicherzustellen, dass sie sicher und mit den konventionellen Behandlungen kompatibel sind.

**C.2. Welche Methoden der ayurvedischen Medizin helfen bei rheumatoider Arthritis?**

Die ayurvedische Medizin, eine traditionelle indische Heilkunst, betrachtet Krankheiten als ein Ungleichgewicht der drei Doshas (Vata, Pitta, Kapha) im Körper. Bei der Behandlung von rheumatoider Arthritis (RA) zielt die ayurvedische Medizin darauf ab, das Gleichgewicht der Doshas wiederherzustellen, Entzündungen zu reduzieren und die allgemeine Gesundheit zu fördern. Hier sind einige häufig verwendete Methoden und Ansätze der ayurvedischen Medizin bei rheumatoider Arthritis:

1. **Kräutermedizin (Rasayana)**: Es gibt verschiedene ayurvedische Kräuterpräparate, die zur Behandlung von rheumatoider Arthritis eingesetzt werden können. Diese Kräuter sollen Entzündungen reduzieren, Schmerzen lindern und die Gelenkfunktion verbessern. Beispiele für solche Kräuter sind Ashwagandha, Guggulu, Triphala und Kurkuma.

2. **Ölbehandlungen (Panchakarma)**: Panchakarma ist eine spezielle Reinigungs- und Entgiftungstherapie in der ayurvedischen Medizin. Bei rheumatoider Arthritis können Ölmassagen (Abhyanga), Dampfbehandlungen (Swedana) und Einläufe (Basti) mit medizinierten Ölen zur Linderung von Schmerzen und Entzündungen eingesetzt werden.

3. **Diät und Lebensstil**: In der ayurvedischen Medizin wird großer Wert auf eine ausgewogene Ernährung und einen gesunden Lebensstil gelegt. Bestimmte Nahrungsmittel und Getränke können empfohlen oder vermieden werden, um das Gleichgewicht der Doshas zu fördern und Entzündungen zu reduzieren. Regelmäßige Bewegung und Yoga-Übungen können ebenfalls hilfreich sein, um die Gelenkfunktion zu verbessern und Stress abzubauen.

4. **Yoga und Meditation**: Yoga-Übungen und Meditation können helfen, den Körper zu stärken, die Flexibilität der Gelenke zu erhöhen und den Geist zu beruhigen. Spezielle Yoga-Posen (Asanas) und Atemübungen (Pranayama) können zur Linderung von RA-Symptomen beitragen und das allgemeine Wohlbefinden fördern.

5. **Ayurvedische Massage (Abhyanga)**: Diese spezielle Massage-Technik mit medizinierten Ölen kann zur Linderung von Schmerzen und Entzündungen beitragen, indem sie den Energiefluss im Körper harmonisiert und die Durchblutung verbessert.

Es ist wichtig zu beachten, dass die ayurvedische Medizin nicht für jeden geeignet ist und nicht als Ersatz für die konventionelle medizinische Behandlung angesehen werden sollte. Vor Beginn einer ayurvedischen Behandlung bei rheumatoider Arthritis oder einer anderen Erkrankung ist es ratsam, einen qualifizierten Ayurveda-Therapeuten oder Arzt zu konsultieren und eine umfassende Diagnose und Behandlungsplanung durchführen zu lassen. Es kann auch hilfreich sein, einen Rheumatologen zu konsultieren, um eine integrative Behandlungsstrategie zu entwickeln.

**C.1.Gibt es phytotherapeutische Medikamente, die bei rheumatoider Arthritis helfen?**

Es gibt einige Pflanzenextrakte und Kräuter, die in der Phytotherapie verwendet werden und denen nachgesagt wird, bei rheumatoider Arthritis (RA) eine gewisse Linderung bieten zu können. Es ist jedoch wichtig zu betonen, dass die wissenschaftliche Evidenz für die Wirksamkeit solcher phytotherapeutischer Ansätze begrenzt ist, und sie sollten nicht als Ersatz für konventionelle medizinische Behandlungen betrachtet werden. Hier sind einige Beispiele:

1. **Kurkuma (Curcuma longa):** Kurkuma enthält Curcumin, das entzündungshemmende Eigenschaften haben kann. Einige Studien haben darauf hingewiesen, dass Curcumin die Symptome von Rheuma lindern kann. Es ist jedoch wichtig zu beachten, dass die Menge an Curcumin in Kurkuma begrenzt ist, und es können hochkonzentrierte Curcumin-Präparate erforderlich sein.

2. **Ingwer (Zingiber officinale):** Ingwer hat entzündungshemmende Eigenschaften und kann zur Linderung von Gelenkschmerzen beitragen. Einige Menschen mit RA berichten von positiven Effekten durch die Einnahme von Ingwer.

3. **Boswellia (Boswellia serrata):** Boswelliasäuren, die in der Rinde von Boswellia-Bäumen vorkommen, haben entzündungshemmende Eigenschaften. Präparate mit Boswellia-Extrakt werden manchmal zur Unterstützung bei entzündlichen Erkrankungen wie RA verwendet.

4. **Weihrauch (Olibanum):** Weihrauchharz wird aus der Rinde von Boswellia-Bäumen gewonnen und wird in der traditionellen Medizin zur Linderung von Entzündungen eingesetzt. Es gibt einige Hinweise darauf, dass Weihrauchpräparate bei RA hilfreich sein könnten.

Es ist wichtig zu betonen, dass diese phytotherapeutischen Ansätze nicht für jeden geeignet sein könnten, und ihre Wirksamkeit kann von Person zu Person variieren. Darüber hinaus sollten solche Präparate nicht ohne Rücksprache mit einem Arzt eingenommen werden, insbesondere wenn sie mit anderen Medikamenten interagieren könnten.

Bevor Sie phytotherapeutische Medikamente in Betracht ziehen, ist es ratsam, dies mit Ihrem behandelnden Arzt zu besprechen, um sicherzustellen, dass sie sicher und mit den konventionellen Behandlungen kompatibel sind.

**C.2. Welche Phytotherapeutika helfen bei rheumatoider Arthritis?**
Die Phytotherapie nutzt Heilpflanzen und ihre Extrakte zur Vorbeugung und Behandlung von Krankheiten. Bei rheumatoider Arthritis (RA) gibt es einige Heilpflanzen und Kräuter, die traditionell zur Linderung von Symptomen eingesetzt werden. Hier sind einige Beispiele für Phytotherapeutika, die bei rheumatoider Arthritis in Betracht gezogen werden könnten:

1. **Kurkuma (Curcuma longa)**: Kurkuma enthält den aktiven Inhaltsstoff Curcumin, der entzündungshemmende und antioxidative Eigenschaften hat. Studien haben gezeigt, dass Curcumin zur Linderung von Entzündungen und Schmerzen bei RA beitragen kann.

2. **Ingwer (Zingiber officinale)**: Ingwer hat entzündungshemmende Eigenschaften und kann zur Linderung von Schmerzen und Entzündungen bei RA beitragen.

3. **Weidenrinde (Salix alba)**: Weidenrinde enthält Salicin, eine Substanz, die ähnlich wie Aspirin wirkt und entzündungshemmende und schmerzlindernde Eigenschaften hat.

4. **Teufelskralle (Harpagophytum procumbens)**: Teufelskralle wird traditionell zur Linderung von Gelenkschmerzen und Entzündungen eingesetzt und kann bei RA zur Verbesserung der Symptome beitragen.

5. **Boswellia (Boswellia serrata)**: Boswellia-Extrakt enthält Boswelliasäuren, die entzündungshemmende Eigenschaften haben und zur Linderung von Gelenkschmerzen und Entzündungen bei RA beitragen können.

6. **Brennnessel (Urtica dioica)**: Brennnesselblätter können entzündungshemmende und schmerzlindernde Eigenschaften haben und zur Behandlung von RA-Symptomen eingesetzt werden.

7. **Johanniskraut (Hypericum perforatum)**: Johanniskraut kann zur Linderung von Nervenschmerzen und Entzündungen bei RA beitragen.

Es ist wichtig zu beachten, dass die Anwendung von Phytotherapeutika bei rheumatoider Arthritis individuell variieren kann und nicht für jeden geeignet ist. Vor der Anwendung von Heilpflanzen oder Kräutern zur Behandlung von RA ist es ratsam, einen Arzt oder Apotheker zu konsultieren, um mögliche Wechselwirkungen mit anderen Medikamenten oder gesundheitlichen Bedingungen zu berücksichtigen. Es ist auch wichtig, qualitativ hochwertige Produkte von vertrauenswürdigen Herstellern zu verwenden und die empfohlenen Dosierungen nicht zu überschreiten.

**Systemischer Lupus erythematodes (SLE)**

## A. Ich habe einen systemischen Lupus erythematodes. Was soll ich tun?

Der systemische Lupus erythematodes (SLE) ist eine komplexe Autoimmunerkrankung, die verschiedene Organe und Gewebe im Körper betreffen kann. Die Behandlung von SLE erfordert eine sorgfältige Betreuung durch einen Facharzt, normalerweise einen Rheumatologen. Hier sind einige allgemeine Empfehlungen, die Ihnen helfen können, den Umgang mit SLE zu erleichtern:

1. **Regelmäßige ärztliche Betreuung:** Halten Sie regelmäßige Termine mit Ihrem Rheumatologen ein, um den Krankheitsverlauf zu überwachen und die Behandlung anzupassen.

2. **Medikamentöse Behandlung:** Folgen Sie den verschriebenen Medikamenten und nehmen Sie diese gemäß den Anweisungen Ihres Arztes ein. Die medikamentöse Behandlung kann entzündungshemmende Medikamente, Immunsuppressiva, Steroide und andere Medikamente umfassen.

3. **Selbstmanagement:** Lernen Sie, Ihre Symptome zu überwachen und mit Ihrem Arzt zu kommunizieren. Notieren Sie Änderungen oder neue Symptome und teilen Sie diese Informationen bei Ihren Arztbesuchen mit.

4. **Gesunde Lebensgewohnheiten:** Achten Sie auf eine gesunde Lebensweise, einschließlich ausgewogener Ernährung, ausreichender Ruhe, regelmäßiger Bewegung und Stressbewältigung.

5. **Sonnenexposition:** Personen mit SLE sollten Sonnenschutzmaßnahmen treffen, da UV-Strahlung bekanntermaßen Schübe auslösen kann. Verwenden Sie Sonnenschutzmittel, bedecken Sie Ihre Haut und tragen Sie Hüte bei Sonneneinstrahlung.

6. **Stressmanagement:** Praktizieren Sie Stressbewältigungstechniken wie Meditation, Yoga oder tiefes Atmen, da Stress Schübe verschlimmern kann.

7. **Gesunde Ernährung:** Eine ausgewogene Ernährung mit ausreichend Nährstoffen kann zur allgemeinen Gesundheit beitragen. Einige Menschen mit SLE berichten von einer Verbesserung ihrer Symptome durch eine entzündungshemmende Ernährung.

8. **Informationssuche:** Informieren Sie sich über SLE und verstehen Sie Ihre Erkrankung. Dies kann Ihnen helfen, besser mit der Krankheit umzugehen und fundierte Entscheidungen über Ihre Gesundheit zu treffen.

Es ist wichtig zu betonen, dass diese allgemeinen Empfehlungen nicht als Ersatz für die individuelle medizinische Betreuung dienen sollten. Jeder Fall von SLE ist einzigartig, und Ihre Behandlung sollte auf Ihre spezifischen Bedürfnisse zugeschnitten sein. Konsultieren Sie regelmäßig Ihren Rheumatologen, um Ihre Symptome zu besprechen und Ihre Behandlung anzupassen.

## B.1. Kann ich die schulmedizinischen Medikamente durch Verfahren der Alternativmedizin oder Naturheilkunde ersetzen, wenn ich einen systemischen Lupus erythematodes habe?

Die schulmedizinischen Medikamente, die von Ärzten zur Behandlung von systemischem Lupus erythematodes (SLE) verschrieben werden, spielen eine entscheidende Rolle bei der Kontrolle von Symptomen, der Verwaltung von Krankheitsaktivität und der Verbesserung der Lebensqualität. Es

gibt keine wissenschaftlichen Beweise dafür, dass Verfahren der Alternativmedizin oder Naturheilkunde schulmedizinische Medikamente bei SLE ersetzen können.

SLE ist eine komplexe Autoimmunerkrankung, die eine sorgfältige medizinische Überwachung und Behandlung erfordert. Die schulmedizinischen Ansätze umfassen oft entzündungshemmende Medikamente, Immunsuppressiva und andere Medikamente, um Symptome zu kontrollieren und die Krankheit zu managen.

Es ist wichtig zu verstehen, dass alternative Ansätze, einschließlich Naturheilkunde und Alternativmedizin, oft nicht ausreichend wissenschaftlich erforscht oder validiert sind, um schulmedizinische Behandlungen zu ersetzen. Es gibt Menschen, die subjektive Verbesserungen durch alternative Ansätze erfahren, aber diese Erfahrungen sind oft individuell unterschiedlich und können nicht verallgemeinert werden.

Es wird dringend empfohlen, dass Sie keine schulmedizinischen Medikamente ohne Absprache mit Ihrem behandelnden Arzt absetzen oder durch alternative Ansätze ersetzen. Die Selbstbehandlung von SLE ohne angemessene medizinische Überwachung kann zu Verschlechterung der Symptome, Krankheitsprogression und anderen gesundheitlichen Komplikationen führen.

Wenn Sie Interesse an alternativen Ansätzen haben, ist es wichtig, diese mit Ihrem Rheumatologen oder einem qualifizierten Gesundheitsdienstleister zu besprechen. Ein integrierter Ansatz, bei dem schulmedizinische Behandlungen und alternative Ansätze sorgfältig koordiniert werden, kann in einigen Fällen angemessen sein. Ihr Arzt kann Ihnen dabei helfen, eine individuell angepasste und sichere Behandlungsstrategie zu entwickeln.

**B.2. Mit welchen Verfahren der Alternativmedizin oder Naturheilkunde kann ich die schulmedizinischen Medikamente ersetzen, wenn ich einen systemischen Lupus erythematodes habe?**

Es ist wichtig zu betonen, dass systemischer Lupus erythematodes (SLE) eine komplexe Autoimmunerkrankung ist, die oft eine sorgfältige medizinische Betreuung erfordert. Schulmedizinische Medikamente werden häufig verschrieben, um Symptome zu kontrollieren und die Krankheitsaktivität zu modulieren. Es gibt keine wissenschaftlichen Belege dafür, dass Alternativmedizin oder Naturheilkunde schulmedizinische Medikamente bei SLE ersetzen können.

Die Schulmedizin bietet Ansätze wie entzündungshemmende Medikamente, Immunsuppressiva und andere Medikamente, um Symptome zu kontrollieren und die Krankheit zu verwalten. Es ist wichtig, dass Sie jede Entscheidung bezüglich Ihrer Behandlung mit Ihrem Rheumatologen oder einem qualifizierten Arzt besprechen, um sicherzustellen, dass Ihre Gesundheitsbedürfnisse angemessen und sicher berücksichtigt werden.

Dennoch gibt es viele Menschen, die sich für ergänzende Ansätze interessieren. Wenn Sie alternative Verfahren oder Naturheilkunde in Betracht ziehen, ist es entscheidend, dies mit Ihrem Arzt abzustimmen. Einige Menschen erleben subjektive Vorteile durch bestimmte Ansätze, aber ihre Wirksamkeit und Sicherheit sind oft nicht ausreichend wissenschaftlich nachgewiesen.

Hier sind einige alternative Ansätze, die von einigen Menschen mit SLE erkundet wurden:

1. **Akupunktur:** Einige Menschen berichten von einer Linderung von Schmerzen und Stresssymptomen durch Akupunktur. Es sollte jedoch als ergänzende Therapie betrachtet werden.

2. **Yoga und Meditation:** Yoga und Meditation können zur Stressbewältigung beitragen und das allgemeine Wohlbefinden fördern. Dies ersetzt jedoch nicht die notwendigen medizinischen Behandlungen.

3. **Ernährungsumstellungen:** Einige Menschen mit SLE berichten von einer Verbesserung ihrer Symptome durch eine gesunde und ausgewogene Ernährung. Eine entzündungshemmende Ernährung kann unterstützend wirken.

4. **Kräutermedizin und Naturheilmittel:** Einige Kräuter und Naturheilmittel werden in alternativen Ansätzen verwendet. Es ist jedoch wichtig, diese mit Ihrem Arzt zu besprechen, da sie Wechselwirkungen mit Medikamenten haben können.

Es ist entscheidend zu betonen, dass alternative Ansätze nicht als Ersatz für schulmedizinische Behandlungen dienen sollten. Jeder Fall von SLE ist einzigartig, und die beste Behandlungsstrategie sollte individuell mit einem qualifizierten Arzt besprochen werden.

**C.1. Gibt es Homöopathische Medikamente, die bei systemischem Lupus erythematodes helfen?**

Es gibt homöopathische Mittel, die bei systemischem Lupus erythematodes (SLE) verwendet werden könnten, aber es ist wichtig zu beachten, dass die Homöopathie als alternative Medizin und ihre Wirksamkeit bei SLE nicht durch wissenschaftliche Studien unterstützt sind. Systemischer Lupus erythematodes ist eine komplexe Autoimmunerkrankung, die eine angemessene medizinische Betreuung erfordert.

Homöopathie basiert auf dem Prinzip der Ähnlichkeit, bei dem stark verdünnte Substanzen verwendet werden, die in höheren Dosen ähnliche Symptome wie die Krankheit verursachen würden. Die Auswahl der homöopathischen Mittel erfolgt oft auf der Grundlage der individuellen Symptome und der gesamten Konstitution der Person.

Einige homöopathische Mittel, die in der Literatur als möglicherweise hilfreich bei SLE genannt werden, könnten Arsenicum album, Apis mellifica, Lachesis, Belladonna, und andere sein. Dennoch ist es wichtig zu betonen, dass diese Empfehlungen auf Erfahrungen von Homöopathen basieren und keine wissenschaftliche Grundlage haben.

Es ist entscheidend zu betonen, dass bei einer komplexen Erkrankung wie systemischem Lupus erythematodes die evidenzbasierte medizinische Betreuung im Vordergrund stehen sollte. Die Behandlung von SLE umfasst oft entzündungshemmende Medikamente, Immunsuppressiva und andere Therapien, um die Symptome zu kontrollieren und die Krankheitsaktivität zu managen.

Wenn jemand homöopathische Mittel in Erwägung zieht, ist es wichtig, dies mit einem qualifizierten Arzt zu besprechen. Eine integrative Herangehensweise, die Homöopathie und konventionelle Medizin kombiniert, sollte unter ärztlicher Aufsicht erfolgen, um sicherzustellen, dass alle Aspekte der Behandlung sicher und effektiv sind.

**C.2. Welche homöopathischen Arzneimittel helfen bei systemischem Lupus erythematodes?**

Die Homöopathie betrachtet jeden Patienten als einzigartig, und die Auswahl homöopathischer Arzneimittel basiert auf einer detaillierten Analyse der individuellen Symptome, der Konstitution und der Krankheitsgeschichte. Es gibt keine spezifischen homöopathischen Arzneimittel, die als Standardbehandlung für den systemischen Lupus erythematodes (SLE) gelten, da die homöopathische Medizin darauf abzielt, das Gleichgewicht der Lebenskraft im Körper zu fördern.

Ein erfahrener Homöopath wird eine ausführliche Anamnese durchführen, um ein individuelles Arzneimittel für Ihre spezifischen Symptome zu verschreiben. Hier sind jedoch einige homöopathische Mittel, die in Betracht gezogen werden könnten, je nach den vorherrschenden Symptomen:

1. **Arsenicum album:** Bei Erschöpfung, Ängstlichkeit, und Unruhe.

2. **Lachesis:** Für Personen, die sich steif fühlen und Schwierigkeiten beim Schlucken haben können. Es könnte für Menschen mit Hautsymptomen nützlich sein.

3. **Apis mellifica:** Bei Schwellungen, Rötungen und Hitzegefühlen. Es könnte auch bei Gelenkbeschwerden helfen.

4. **Belladonna:** Wenn plötzliche Schübe von Hitze, Röte und Entzündungen auftreten.

5. **Natrum muriaticum:** Bei Mundgeschwüren, Hautausschlägen und emotionaler Empfindlichkeit.

Es ist wichtig zu betonen, dass die Homöopathie als ergänzende Therapie betrachtet werden sollte und nicht als Ersatz für schulmedizinische Behandlungen. SLE erfordert oft eine umfassende medizinische Betreuung durch einen Rheumatologen. Bevor Sie homöopathische Mittel anwenden, ist es ratsam, dies mit Ihrem behandelnden Arzt zu besprechen, um sicherzustellen, dass diese sicher und effektiv in Ihren Gesamtbehandlungsplan integriert werden können. Selbstmedikation sollte vermieden werden, und die individuelle Beratung durch einen erfahrenen Homöopathen ist entscheidend.

**C.1. Gibt es Methoden der ayurvedischen Medizin, die bei systemischem Lupus erythematodes helfen?**

Systemischer Lupus erythematodes (SLE) ist eine komplexe Autoimmunerkrankung, und es gibt keine wissenschaftlichen Beweise dafür, dass ayurvedische Medizin eine Heilung bietet. Es gibt jedoch einige ayurvedische Ansätze, die potenziell dazu dienen könnten, die Symptome von SLE zu lindern oder das allgemeine Wohlbefinden zu verbessern. Es ist wichtig zu beachten, dass diese Ansätze nicht als Ersatz für die konventionelle medizinische Behandlung betrachtet werden sollten.

Einige ayurvedische Methoden, die in Erwägung gezogen werden können, sind:

1. **Panchakarma:** Dies ist eine Reihe von Reinigungsprozeduren in der ayurvedischen Medizin, die darauf abzielen, den Körper zu entgiften. Panchakarma kann auf die Reduzierung von Toxinen abzielen und das Gleichgewicht der Doshas im Körper wiederherstellen.

2. **Kräuterpräparate:** In der ayurvedischen Medizin werden verschiedene Kräuter für ihre entzündungshemmenden und immunmodulierenden Eigenschaften geschätzt. Ein erfahrener Ayurveda-Praktiker kann individuell angepasste Kräuterpräparate empfehlen.

3. **Ernährung:** Die Ayurveda-Behandlung betont die Bedeutung einer ausgewogenen Ernährung, die auf die individuelle Konstitution abgestimmt ist. Dies könnte helfen, das Gleichgewicht der Doshas zu unterstützen.

4. **Yoga und Meditation:** Diese Praktiken werden oft als Mittel zur Stressreduktion und Förderung des allgemeinen Wohlbefindens empfohlen.

Es ist wichtig zu beachten, dass die ayurvedischen Ansätze bei SLE nicht als Hauptbehandlung dienen sollten. Die konventionelle medizinische Betreuung bleibt entscheidend, und die Behandlung von SLE erfordert oft Medikamente wie entzündungshemmende Medikamente, Immunsuppressiva und andere Therapien.

Vor der Anwendung von ayurvedischen Methoden ist es ratsam, dies mit Ihrem behandelnden Arzt zu besprechen. Eine integrative Herangehensweise, die sowohl ayurvedische als auch konventionelle Medizin kombiniert, sollte unter ärztlicher Aufsicht erfolgen, um sicherzustellen, dass alle Aspekte der Behandlung sicher und effektiv sind.

**C.2. Welche Methoden der ayurvedischen Medizin helfen bei systemischem Lupus erythematodes?**
In der ayurvedischen Medizin wird systemischer Lupus erythematodes (SLE) als eine Störung des Gleichgewichts der Doshas (Vata, Pitta, Kapha) und des Agni (Verdauungsfeuers) betrachtet. Die ayurvedische Behandlung zielt darauf ab, die individuelle Konstitution zu harmonisieren und das Gleichgewicht der Doshas wiederherzustellen. Es ist wichtig zu beachten, dass Ayurveda als ganzheitlicher Ansatz betrachtet wird und die Behandlung auf die individuellen Symptome und Bedürfnisse des Patienten abgestimmt sein sollte. Hier sind einige Methoden der ayurvedischen Medizin, die bei SLE in Betracht gezogen werden können:

1. **Kräutermedizin:** Ein qualifizierter Ayurveda-Praktiker kann individuell angepasste Kräuterformeln verschreiben, um das Gleichgewicht der Doshas zu fördern und entzündungshemmende Eigenschaften zu unterstützen. Kräuter wie Ashwagandha, Guduchi und Shatavari könnten in Betracht gezogen werden.

2. **Diät nach den Prinzipien des Ayurveda:** Eine ausgewogene Ernährung, die auf die individuelle Konstitution abgestimmt ist, kann dazu beitragen, das Gleichgewicht der Doshas zu unterstützen. Die Betonung liegt auf nahrhaften Lebensmitteln, die gut verdaulich sind.

3. **Panchakarma:** Panchakarma ist eine Reihe von Reinigungs- und Entgiftungsverfahren, die darauf abzielen, die Doshas zu balancieren. Dazu gehören Therapien wie Ölmassagen (Abhyanga), Dampfbäder (Swedana), Einläufe (Basti) und andere.

4. **Yoga und Pranayama:** Spezifische Yogaübungen und Atemtechniken können dazu beitragen, die Flexibilität zu verbessern, Stress abzubauen und das allgemeine Wohlbefinden zu fördern.

5. **Ayurvedische Lebensstilrichtlinien:** Ein ayurvedischer Lebensstil betont das Gleichgewicht zwischen Arbeit und Ruhe, regelmäßige Schlafenszeiten, positive emotionale Gesundheit und andere Lebensgewohnheiten.

Es ist wichtig zu betonen, dass Ayurveda nicht als Ersatz, sondern als unterstützende Therapie zu schulmedizinischen Ansätzen betrachtet werden sollte. Konsultieren Sie einen qualifizierten Ayurveda-Praktiker, um eine umfassende Diagnose durchzuführen und einen individuell angepassten Behandlungsplan zu erstellen. Informieren Sie auch Ihren Rheumatologen über jegliche ayurvedische Ansätze, um sicherzustellen, dass sie sicher und mit anderen medizinischen Maßnahmen kompatibel sind. Die Integration von Ayurveda sollte in Absprache mit beiden Fachleuten erfolgen.

**C.1. Gibt es Phytotherapeutika, die bei systemischem Lupus erythematodes helfen?**

Die phytotherapeutische Medizin, die auf pflanzlichen Wirkstoffen basiert, umfasst verschiedene Pflanzenextrakte und Kräuter, die möglicherweise entzündungshemmende und immunmodulierende Eigenschaften haben. Es gibt jedoch begrenzte wissenschaftliche Beweise für die Wirksamkeit von

Phytotherapie bei systemischem Lupus erythematodes (SLE), einer komplexen Autoimmunerkrankung.

Einige Pflanzenextrakte und Kräuter, die in der Phytotherapie verwendet werden könnten und die entzündungshemmende Eigenschaften haben, sind beispielsweise:

1. **Kurkuma (Curcuma longa):** Enthält Curcumin, das entzündungshemmende Eigenschaften haben kann.

2. **Grüner Tee:** Enthält Polyphenole, die antioxidative und möglicherweise entzündungshemmende Wirkungen haben können.

3. **Boswellia serrata:** Enthält Boswelliasäuren, die entzündungshemmende Eigenschaften haben können.

4. **Ginkgo biloba:** Kann antioxidative Eigenschaften haben, die das Immunsystem unterstützen.

Es ist wichtig zu betonen, dass diese phytotherapeutischen Ansätze nicht als Ersatz für die konventionelle medizinische Behandlung von SLE betrachtet werden sollten. Die Behandlung von SLE erfordert oft entzündungshemmende Medikamente, Immunsuppressiva und andere Therapien, um die Symptome zu kontrollieren und die Krankheitsaktivität zu managen.

Bevor Sie phytotherapeutische Medikamente in Erwägung ziehen, ist es ratsam, dies mit Ihrem behandelnden Arzt zu besprechen. Einige Pflanzenextrakte können Wechselwirkungen mit Medikamenten haben oder unerwünschte Nebenwirkungen verursachen. Die Integration von Phytotherapie in die Behandlung sollte unter Anleitung eines qualifizierten Gesundheitsdienstleisters erfolgen.

**C.2. Welche Phytotherapeutika helfen bei systemischem Lupus erythematodes?**

Die Verwendung von Phytotherapeutika (Heilpflanzen) bei systemischem Lupus erythematodes (SLE) sollte immer in Absprache mit Ihrem Rheumatologen oder einem qualifizierten Gesundheitsdienstleister erfolgen. Einige Pflanzen werden aufgrund ihrer potenziellen entzündungshemmenden oder immunmodulierenden Eigenschaften in Betracht gezogen, jedoch ist die Forschung in diesem Bereich begrenzt. Hier sind einige Heilpflanzen, die möglicherweise bei SLE unterstützend wirken könnten:

1. **Kurkuma (Curcuma longa):** Kurkuma enthält den Wirkstoff Curcumin, der entzündungshemmende Eigenschaften haben kann. Es wird oft als Nahrungsergänzungsmittel eingenommen.

2. **Ingwer (Zingiber officinale):** Ingwer hat entzündungshemmende und antioxidative Eigenschaften. Es kann in der Küche verwendet oder als Nahrungsergänzung eingenommen werden.

3. **Ginkgo biloba:** Ginkgo biloba hat antioxidative Eigenschaften und könnte die Durchblutung verbessern. Einige Menschen mit SLE berichten von einer Verbesserung ihrer Symptome.

4. **Grüner Tee (Camellia sinensis):** Grüner Tee ist reich an Antioxidantien, die entzündungshemmende Wirkungen haben können.

5. **Astragalus (Astragalus membranaceus):** Astragalus wird in der traditionellen chinesischen Medizin verwendet und könnte immunmodulierende Eigenschaften haben.

6. **Mariendistel (Silybum marianum):** Mariendistel kann leberschützende Eigenschaften haben und wird manchmal bei Autoimmunerkrankungen eingesetzt.

7. **Boswellia (Boswellia serrata):** Boswellia ist eine Pflanze, die harzige Extrakte produziert, die entzündungshemmende Eigenschaften haben können.

8. **Teufelskralle (Harpagophytum procumbens):** Teufelskralle wird traditionell zur Linderung von Gelenk- und Muskelschmerzen verwendet und könnte entzündungshemmende Eigenschaften haben.

Es ist wichtig zu betonen, dass die wissenschaftlichen Erkenntnisse über die Wirksamkeit von Heilpflanzen bei SLE begrenzt sind. Vor der Anwendung von Phytotherapeutika sollten Sie daher mit Ihrem Rheumatologen sprechen, um sicherzustellen, dass sie sicher und mit anderen Medikamenten kompatibel sind. Darüber hinaus sollten Heilpflanzen nicht als Ersatz für die von Ihrem Arzt verschriebenen Medikamente betrachtet werden, sondern als ergänzende Maßnahme in Absprache mit Ihrem medizinischen Fachdienstleister.

**Granulomatose mit Polyangiitis (GPA)**

**A. Ich habe eine Granulomatose mit Polyangiitis. Was soll ich tun?**

Die Granulomatose mit Polyangiitis (GPA), früher als Wegener-Granulomatose bekannt, ist eine seltene Autoimmunerkrankung, die die Blutgefäße betrifft und zu Entzündungen in verschiedenen Organen führen kann. Die Behandlung von GPA erfordert in der Regel eine enge Zusammenarbeit mit einem Rheumatologen oder einem Spezialisten für Immunologie. Hier sind einige allgemeine Empfehlungen, die Ihnen helfen können, den Umgang mit GPA zu erleichtern:

1. **Regelmäßige ärztliche Betreuung:** Halten Sie regelmäßige Termine mit Ihrem Rheumatologen oder dem behandelnden Arzt ein, um den Verlauf der Krankheit zu überwachen und die Behandlung anzupassen.

2. **Medikamentöse Behandlung:** Die meisten Menschen mit GPA benötigen immunsuppressive Medikamente, um die Entzündung zu kontrollieren und das Immunsystem zu regulieren. Zu den häufig verwendeten Medikamenten gehören Glukokortikoide (wie Prednison) und Immunsuppressiva wie Cyclophosphamid oder Rituximab.

3. **Selbstmanagement:** Lernen Sie, Ihre Symptome zu überwachen und mit Ihrem Arzt zu kommunizieren. Notieren Sie Änderungen oder neue Symptome und teilen Sie diese Informationen bei Ihren Arztbesuchen mit.

4. **Gesunde Lebensgewohnheiten:** Achten Sie auf eine ausgewogene Ernährung, ausreichend Schlaf, regelmäßige Bewegung und Stressmanagement, um Ihre allgemeine Gesundheit zu unterstützen.

5. **Infektionsprävention:** Aufgrund der immunsuppressiven Therapie haben Menschen mit GPA ein erhöhtes Risiko für Infektionen. Beachten Sie Hygienemaßnahmen, um Infektionen zu vermeiden, und informieren Sie Ihren Arzt, wenn Sie Anzeichen einer Infektion haben.

6. **Rauchverzicht:** Das Rauchen kann den Verlauf von GPA negativ beeinflussen. Wenn Sie Raucher sind, erwägen Sie ernsthaft, mit dem Rauchen aufzuhören.

7. **Unterstützung suchen:** Der Umgang mit einer chronischen Erkrankung kann emotional belastend sein. Suchen Sie Unterstützung von Familie, Freunden oder professionellen Beratern, um Ihre emotionalen Bedürfnisse zu bewältigen.

8. **Informierte Entscheidungen treffen:** Informieren Sie sich über GPA, um ein besseres Verständnis für Ihre Erkrankung zu entwickeln. Dies kann Ihnen helfen, fundierte Entscheidungen über Ihre Gesundheit zu treffen.

**B.1. Kann ich die schulmedizinischen Medikamente durch Verfahren der Alternativmedizin oder Naturheilkunde ersetzen, wenn ich eine Granulomatose mit Polyangiitis habe?**

Es wird dringend davon abgeraten, schulmedizinische Medikamente eigenmächtig durch Verfahren der Alternativmedizin oder Naturheilkunde zu ersetzen, insbesondere bei ernsthaften Erkrankungen wie der Granulomatose mit Polyangiitis (GPA). GPA ist eine Autoimmunerkrankung, bei der das Immunsystem körpereigenes Gewebe angreift, und die schulmedizinische Behandlung zielt darauf ab, die überaktive Immunantwort zu kontrollieren und Entzündungen zu reduzieren.

Die schulmedizinische Therapie für GPA umfasst normalerweise Immunsuppressiva und Glukokortikoide, die dazu beitragen, das Fortschreiten der Krankheit zu verhindern und Symptome zu lindern. Diese Medikamente sind sorgfältig auf die individuellen Bedürfnisse des Patienten abgestimmt, und ihre Wirkung muss regelmäßig überwacht werden.

Alternativmedizin und Naturheilkunde können in einigen Fällen als unterstützende Maßnahmen in Betracht gezogen werden, sollten jedoch nicht die Hauptbehandlung ersetzen. Ein integrativer Ansatz, bei dem schulmedizinische Therapie und alternative Methoden kombiniert werden, kann unter Aufsicht eines qualifizierten Gesundheitsdienstleisters in Betracht gezogen werden. Es ist wichtig, dass Sie alle Änderungen in Ihrer Behandlung mit Ihrem behandelnden Arzt besprechen, um sicherzustellen, dass Ihr Gesundheitszustand angemessen überwacht wird.

Eigenmächtiges Absetzen oder Ersetzen von verschriebenen Medikamenten kann schwerwiegende Folgen haben und das Risiko von Krankheitsschüben und Komplikationen erhöhen. Ihr Arzt ist der beste Ansprechpartner, um gemeinsam mit Ihnen die besten Entscheidungen für Ihre Gesundheit zu treffen.

**B.2. Mit welchen Verfahren der Alternativmedizin oder Naturheilkunde kann ich die schulmedizinischen Medikamente ersetzen, wenn ich eine Granulomatose mit Polyangiitis habe?**

Es ist wichtig zu betonen, dass diese Empfehlungen allgemeiner Natur sind und nicht als Ersatz für die individuelle medizinische Betreuung dienen sollten. Jeder Fall von GPA ist einzigartig, und Ihre Behandlung sollte auf Ihre spezifischen Bedürfnisse zugeschnitten sein. Konsultieren Sie regelmäßig Ihren Rheumatologen, um den Verlauf der Erkrankung zu besprechen und die Behandlung anzupassen.

Es ist äußerst wichtig zu betonen, dass die Granulomatose mit Polyangiitis (GPA) eine ernste und potenziell lebensbedrohliche Autoimmunerkrankung ist. Die schulmedizinische Behandlung, die normalerweise Medikamente wie Immunsuppressiva und Glukokortikoide umfasst, zielt darauf ab, die überaktive Immunantwort zu kontrollieren und Entzündungen zu reduzieren. Die schulmedizinische Behandlung ist entscheidend, um das Fortschreiten der Krankheit zu verhindern und mögliche Komplikationen zu minimieren.

Es wird nicht empfohlen, schulmedizinische Medikamente eigenmächtig durch Verfahren der Alternativmedizin oder Naturheilkunde zu ersetzen. Diese alternativen Ansätze können möglicherweise als ergänzende Therapien in Betracht gezogen werden, aber sie sollten immer unter Aufsicht eines qualifizierten Arztes erfolgen, der sich mit Autoimmunerkrankungen auskennt.

Einige Menschen mit GPA erkunden alternative Ansätze, wie beispielsweise:

1. **Ernährungsoptimierung:** Einige Menschen mit Autoimmunerkrankungen berichten von Verbesserungen ihrer Symptome durch eine entzündungshemmende Ernährung. Dies kann den Verzehr von Lebensmitteln einschließen, die reich an Omega-3-Fettsäuren, Antioxidantien und entzündungshemmenden Verbindungen sind.

2. **Akupunktur:** Akupunktur wird manchmal als unterstützende Maßnahme zur Linderung von Symptomen wie Schmerzen und Ermüdung in Betracht gezogen.

3. **Entspannungstechniken:** Stressmanagement durch Methoden wie Meditation, Yoga oder Atemübungen kann für einige Menschen vorteilhaft sein.

4. **Kräutermedizin:** Einige Pflanzen und Kräuter werden in verschiedenen Traditionen für ihre entzündungshemmenden Eigenschaften geschätzt. Es ist jedoch wichtig zu betonen, dass die Anwendung von Kräutern mit einem qualifizierten Kräuterkundigen oder Arzt abgestimmt werden sollte, um mögliche Wechselwirkungen und unerwünschte Effekte zu vermeiden.

Es ist von entscheidender Bedeutung, dass Sie jede Entscheidung bezüglich Ihrer Gesundheitsversorgung mit Ihrem behandelnden Arzt besprechen. Eigenmächtiges Absetzen oder Ersetzen von verschriebenen Medikamenten kann schwerwiegende Folgen haben und das Krankheitsmanagement beeinträchtigen. Ihr Arzt kann Ihnen dabei helfen, einen integrativen Ansatz zu entwickeln, der schulmedizinische Therapie mit unterstützenden Maßnahmen aus der Alternativmedizin kombiniert, wenn dies für Sie geeignet ist.

**C.1. Gibt es Homöopathische Medikamente, die bei Granulomatose mit Polyangiitis helfen?**

Es gibt keine wissenschaftlichen Beweise für die Wirksamkeit von homöopathischen Medikamenten bei der Granulomatose mit Polyangiitis (GPA), einer ernsten und oft lebensbedrohlichen Vaskulitis-Erkrankung. Homöopathie basiert auf dem Prinzip der Ähnlichkeit, bei dem stark verdünnte Substanzen verwendet werden, die in höheren Dosen ähnliche Symptome wie die Krankheit verursachen würden. Obwohl einige Menschen auf homöopathische Behandlungen positiv reagieren können, basiert dies oft auf individuellen Erfahrungen und nicht auf wissenschaftlichen Studien.

Die Behandlung der Granulomatose mit Polyangiitis erfordert normalerweise aggressive medizinische Maßnahmen, darunter entzündungshemmende Medikamente wie Glukokortikoide und Immunsuppressiva. In einigen Fällen können auch biologische Medikamente erforderlich sein.

Es ist wichtig zu betonen, dass homöopathische Mittel nicht als Ersatz für die konventionelle medizinische Behandlung bei GPA betrachtet werden sollten. Die Behandlung sollte unter der Anleitung von Fachärzten erfolgen, die mit den neuesten medizinischen Erkenntnissen und Leitlinien vertraut sind.

Bevor Sie alternative Therapien wie Homöopathie in Erwägung ziehen, ist es ratsam, dies mit Ihrem behandelnden Arzt zu besprechen. Eine umfassende medizinische Betreuung ist bei GPA von entscheidender Bedeutung, um die bestmöglichen Ergebnisse zu erzielen und Komplikationen zu verhindern.

**C.2. Welche homöopathischen Arzneimittel helfen bei Granulomatose mit Polyangiitis?**

Es gibt keine wissenschaftlichen Beweise oder klinischen Studien, die die Wirksamkeit von homöopathischen Arzneimitteln bei der Granulomatose mit Polyangiitis (GPA) belegen. Homöopathie basiert auf dem Prinzip "Similia similibus curentur" (Ähnliches wird mit Ähnlichem geheilt) und verwendet stark verdünnte Substanzen, um den Körper anzuregen, sich selbst zu heilen.

Es ist wichtig zu betonen, dass Homöopathie nicht als Ersatz für die schulmedizinische Behandlung bei ernsthaften Erkrankungen wie GPA betrachtet werden sollte. Die schulmedizinische Behandlung, die in der Regel von einem Rheumatologen oder einem Spezialisten für Immunologie verschrieben wird, ist entscheidend, um die Entzündung zu kontrollieren und das Fortschreiten der Krankheit zu verhindern.

Wenn Sie alternative Ansätze wie Homöopathie in Erwägung ziehen, ist es ratsam, dies mit Ihrem behandelnden Arzt zu besprechen. Ein erfahrener Arzt kann Sie über mögliche Wechselwirkungen mit anderen Medikamenten informieren und sicherstellen, dass Ihre Gesundheit nicht gefährdet wird.

Es ist wichtig, eine ganzheitliche Herangehensweise zu verfolgen, die schulmedizinische Ansätze mit alternativen Methoden kombiniert. Jeder Mensch ist individuell, und die Behandlung sollte auf Ihre spezifischen Bedürfnisse abgestimmt sein. Verlassen Sie sich bei der Behandlung von GPA auf die fundierten Entscheidungen Ihres medizinischen Teams.

**C.1. Gibt es Methoden der ayurvedischen Medizin, die bei Granulomatose mit Polyangiitis helfen?**

Die Granulomatose mit Polyangiitis (GPA) ist eine schwere autoimmunbedingte Vaskulitis-Erkrankung, bei der Blutgefäße entzündet sind. In Bezug auf die ayurvedische Medizin gibt es keine spezifischen wissenschaftlichen Beweise oder standardisierten ayurvedischen Behandlungsprotokolle für die GPA.

Ayurveda ist ein ganzheitliches System, das die Harmonie von Körper, Geist und Seele betont. Ein erfahrener Ayurveda-Praktiker könnte individuelle Ansätze zur Unterstützung des allgemeinen Wohlbefindens vorschlagen, könnte jedoch keine spezifische Heilung für GPA versprechen. Hier sind einige allgemeine ayurvedische Ansätze, die bei Autoimmunerkrankungen in Erwägung gezogen werden könnten:

1. **Panchakarma:** Eine Reihe von Reinigungsprozeduren, die darauf abzielen, den Körper zu entgiften und das Gleichgewicht der Doshas (Vata, Pitta, Kapha) zu harmonisieren.

2. **Kräutermedizin:** Ayurveda verwendet verschiedene Kräuter für ihre entzündungshemmenden und immunmodulierenden Eigenschaften. Ein erfahrener Ayurveda-Praktiker kann individuell angepasste Kräuterrezepturen empfehlen.

3. **Ernährung:** Eine auf die individuelle Konstitution abgestimmte Ernährung kann helfen, das Gleichgewicht der Doshas aufrechtzuerhalten.

4. **Yoga und Meditation:** Diese Praktiken können dazu beitragen, Stress abzubauen und das allgemeine Wohlbefinden zu fördern.

Es ist wichtig zu betonen, dass die ayurvedischen Ansätze bei GPA nicht als Hauptbehandlung dienen sollten. Die Behandlung von GPA erfordert in der Regel entzündungshemmende Medikamente, Immunsuppressiva und manchmal sogar biologische Medikamente.

Bevor Sie alternative Therapien wie Ayurveda in Betracht ziehen, ist es ratsam, dies mit Ihrem behandelnden Arzt zu besprechen. Eine integrative Herangehensweise, die Ayurveda und konventionelle Medizin kombiniert, sollte unter Anleitung eines qualifizierten Gesundheitsdienstleisters erfolgen, um sicherzustellen, dass alle Aspekte der Behandlung sicher und effektiv sind.

**C.2. Welche Methoden der ayurvedischen Medizin helfen bei Granulomatose mit Polyangiitis?**
Die ayurvedische Medizin betrachtet den Menschen als Ganzes und strebt nach dem Gleichgewicht der drei Doshas (Vata, Pitta, Kapha), um die Gesundheit zu fördern. Es ist wichtig zu beachten, dass es begrenzte wissenschaftliche Beweise oder klinische Studien zur Anwendung von ayurvedischen Methoden bei der Granulomatose mit Polyangiitis (GPA) gibt. Die schulmedizinische Behandlung, die von einem Rheumatologen oder Immunologen verschrieben wird, ist in der Regel notwendig, um die Symptome zu lindern und das Fortschreiten der Krankheit zu kontrollieren.

Dennoch können einige ayurvedische Ansätze möglicherweise als unterstützende Maßnahmen in Betracht gezogen werden. Hier sind einige Aspekte der Ayurveda, die in Betracht gezogen werden könnten:

1. **Kräutermedizin:** Ein erfahrener Ayurveda-Praktiker kann eine individuell angepasste Mischung aus ayurvedischen Kräutern empfehlen, die darauf abzielt, das Gleichgewicht im Körper wiederherzustellen und spezifische Symptome zu lindern.

2. **Ayurvedische Ernährung:** Die Ayurveda-Behandlung beinhaltet oft diätetische Empfehlungen, um das Gleichgewicht der Doshas zu fördern. Ein Ayurveda-Experte kann spezifische Nahrungsmittel empfehlen, die als förderlich für Ihre individuelle Konstitution angesehen werden.

3. **Panchakarma:** Dies ist eine Reinigungstherapie im Ayurveda, die darauf abzielt, Toxine aus dem Körper zu entfernen und das Gleichgewicht der Doshas wiederherzustellen. Panchakarma kann verschiedene Techniken wie Ölmassagen, Dampfbäder und Kräuterdetoxifikation einschließen.

4. **Yoga und Meditation:** Yoga und Meditation sind integraler Bestandteil des Ayurveda und können dazu beitragen, Stress zu reduzieren, das allgemeine Wohlbefinden zu verbessern und das Gleichgewicht im Körper zu fördern.

Bevor Sie sich für ayurvedische Methoden entscheiden, sollten Sie dies mit Ihrem Rheumatologen besprechen, um sicherzustellen, dass sie sicher und mit Ihrer aktuellen medizinischen Behandlung kompatibel sind. Ayurveda sollte nicht als Ersatz, sondern als Ergänzung zur schulmedizinischen Behandlung betrachtet werden. Es ist wichtig, dass ayurvedische Ansätze unter der Aufsicht qualifizierter Fachleute durchgeführt werden.

**C.1. Gibt es phytotherapeutische Medikamente, die bei Granulomatose mit Polyangiitis helfen?**
In Bezug auf die Granulomatose mit Polyangiitis (GPA) gibt es keine spezifischen phytotherapeutischen Medikamente, die als Hauptbehandlung für diese Autoimmunerkrankung empfohlen werden können. Die Hauptbehandlung für GPA umfasst normalerweise entzündungshemmende Medikamente wie Glukokortikoide und Immunsuppressiva, um die Entzündung zu kontrollieren und das Immunsystem zu modulieren.

Phytotherapie, auch als Kräutermedizin bekannt, bezieht sich auf die Verwendung von Pflanzen und Pflanzenextrakten zur Vorbeugung und Behandlung von Krankheiten. Es gibt einige Pflanzen, die entzündungshemmende und immunmodulierende Eigenschaften haben könnten, aber ihre Anwendung bei GPA sollte unter ärztlicher Aufsicht erfolgen.

Beispielhafte Pflanzenextrakte, die in der Phytotherapie verwendet werden könnten und entzündungshemmende Eigenschaften haben, sind:

1. **Boswellia serrata:** Enthält Boswelliasäuren, die entzündungshemmende Eigenschaften haben können.

2. **Kurkuma (Curcuma longa):** Enthält Curcumin, das entzündungshemmende Eigenschaften haben kann.

3. **Ingwer (Zingiber officinale):** Enthält Gingerole mit möglichen entzündungshemmenden Wirkungen.

Es ist wichtig zu betonen, dass die Verwendung von Phytotherapie bei GPA nicht als Ersatz für die konventionelle medizinische Behandlung dienen sollte. Die standardmäßige medizinische Betreuung, die entzündungshemmende Medikamente und Immunsuppressiva einschließt, bleibt entscheidend für die Kontrolle der Erkrankung und die Verhinderung von Komplikationen.

Vor der Anwendung von Phytotherapie ist es ratsam, dies mit Ihrem behandelnden Arzt zu besprechen. Einige Pflanzenextrakte können Wechselwirkungen mit Medikamenten haben oder unerwünschte

Nebenwirkungen verursachen. Die Integration von Phytotherapie sollte unter Anleitung eines qualifizierten Gesundheitsdienstleisters erfolgen.

**C.2. Welche Phytotherapeutika helfen bei Granulomatose mit Polyangiitis?**

Es gibt begrenzte wissenschaftliche Beweise oder klinische Studien über die spezifische Anwendung von Phytotherapeutika (Heilpflanzen) bei der Granulomatose mit Polyangiitis (GPA). Die schulmedizinische Behandlung, die von einem Rheumatologen oder Immunologen verschrieben wird, bleibt der Hauptbestandteil bei der Bewältigung von GPA. Jedoch gibt es einige Heilpflanzen, die traditionell für ihre entzündungshemmenden oder immunmodulierenden Eigenschaften bekannt sind. Es ist wichtig zu beachten, dass diese Informationen auf traditionellen Ansätzen basieren und keine Garantie für Wirksamkeit oder Sicherheit darstellen. Bevor Sie Heilpflanzen einnehmen, sollten Sie dies mit Ihrem Arzt besprechen, um sicherzustellen, dass sie sicher und mit Ihrer aktuellen medizinischen Behandlung kompatibel sind. Hier sind einige Heilpflanzen, die in der traditionellen Anwendung enthalten sein könnten:

1. **Kurkuma (Curcuma longa):** Kurkuma enthält Curcumin, das entzündungshemmende Eigenschaften haben kann. Es wird in einigen Kulturen für seine potenziellen gesundheitsfördernden Eigenschaften geschätzt.

2. **Ingwer (Zingiber officinale):** Ingwer hat entzündungshemmende Eigenschaften und kann bei einigen Menschen zur Linderung von Entzündungen beitragen.

3. **Weihrauch (Boswellia serrata):** Weihrauchextrakte können entzündungshemmende Eigenschaften haben und wurden in einigen traditionellen Medizinsystemen verwendet.

4. **Grüner Tee (Camellia sinensis):** Grüner Tee enthält Polyphenole mit antioxidativen und entzündungshemmenden Wirkungen.

Es ist wichtig zu betonen, dass Heilpflanzen nicht die schulmedizinische Behandlung ersetzen sollten. Die Dosierung und Anwendung von Heilpflanzen können je nach individueller Gesundheitssituation variieren, und es ist ratsam, dies mit einem Fachmann zu besprechen. Darüber hinaus können Wechselwirkungen mit anderen Medikamenten auftreten, weshalb es wichtig ist, diese mit Ihrem Arzt zu besprechen.
